# Schizophrenia Synaptic Pathology and Antipsychotic Treatment in the Framework of Oxidative and Mitochondrial Dysfunction: Translational Highlights for the Clinics and Treatment

**DOI:** 10.3390/antiox12040975

**Published:** 2023-04-21

**Authors:** Giuseppe De Simone, Benedetta Mazza, Licia Vellucci, Annarita Barone, Mariateresa Ciccarelli, Andrea de Bartolomeis

**Affiliations:** 1 Section of Psychiatry, Laboratory of Translational and Molecular Psychiatry and Unit of Treatment-Resistant Psychosis, Department of Neuroscience, Reproductive Sciences, and Dentistry, University Medical School of Naples “Federico II”, Via Pansini 5, 80131 Naples, Italy; 2 UNESCO Chair on Health Education and Sustainable Development, University of Naples “Federico II”, 80131 Naples, Italy

**Keywords:** antipsychotics, mitochondria, antioxidants, dopamine, synapse, treatment resistant, schizophrenia, glutamate, postsynaptic density, glutathione

## Abstract

Schizophrenia is a worldwide mental illness characterized by alterations at dopaminergic and glutamatergic synapses resulting in global dysconnectivity within and between brain networks. Impairments in inflammatory processes, mitochondrial functions, energy expenditure, and oxidative stress have been extensively associated with schizophrenia pathophysiology. Antipsychotics, the mainstay of schizophrenia pharmacological treatment and all sharing the common feature of dopamine D2 receptor occupancy, may affect antioxidant pathways as well as mitochondrial protein levels and gene expression. Here, we systematically reviewed the available evidence on antioxidants’ mechanisms in antipsychotic action and the impact of first- and second-generation compounds on mitochondrial functions and oxidative stress. We further focused on clinical trials addressing the efficacy and tolerability of antioxidants as an augmentation strategy of antipsychotic treatment. EMBASE, Scopus, and Medline/PubMed databases were interrogated. The selection process was conducted in respect of the Preferred Reporting Items for Systematic Reviews and Meta-Analyses (PRISMA) criteria. Several mitochondrial proteins involved in cell viability, energy metabolism, and regulation of oxidative systems were reported to be significantly modified by antipsychotic treatment with differences between first- and second-generation drugs. Finally, antioxidants may affect cognitive and psychotic symptoms in patients with schizophrenia, and although the evidence is only preliminary, the results indicate that further studies are warranted.

## 1. Introduction

The “quantal” alteration at both structural and functional levels of schizophrenia pathophysiology is believed to reside in the dynamics of synapse microdomains with specific involvement of dopaminergic and glutamatergic sites and a dysfunctional dopamine-glutamate interaction [1,2,3,4,5]. Alterations at the synaptic level can impair integrative processes on brain macroscale networks, ensuing in a global dysconnectivity pattern, which may be associated, at least in part, with the pathophysiology and clinics of the disorder [4,6,7].

Antipsychotics are the cornerstone of the pharmacological treatment of schizophrenia. Despite the complexity of their receptor profiles and individual receptor affinity, they all share a common feature believed to be the core of antipsychotic action: the occupancy of the dopamine D2 receptor (D2R) [8]. This feature intersects coherently with one major landmark of schizophrenia pathophysiology, which is the increased release of dopamine in the striatum as demonstrated through dynamic positron emission tomography (PET) by means of ^11^C-raclopride [9,10] and increased dopamine capacity detected via 3,4-dihydroxy-6-[^18^F]fluoro-l-phenylalanine (^18^F-DOPA) PET [11] in psychotic patients compared to healthy controls. Antipsychotics can be divided into first- (introduced in the 1950s and 1960s) and second-generation (introduced in the 1990s with the exception of clozapine introduced first in 1970s, withdrawn, and the reintroduced in therapy again), also known as typical and atypical antipsychotics respectively, based on clinical features and receptor signature [12]. First-generation antipsychotics (e.g., haloperidol, chlorpromazine, perphenazine, etc.) have a medium-high D2R affinity [12]. Second-generation antipsychotics (e.g., clozapine, risperidone, olanzapine, quetiapine, paliperidone, lurasidone, etc.) have a multimodal receptor profile with few exceptions (i.e., amisulpride and other benzamides) and a low ratio of D2R/serotonin 2A receptors (5HT_2A_R) affinity, responsible for the low liability to induce extrapyramidal symptoms (EPS) and believed to be partially associated to the moderate effect on negative symptoms [12,13]. Clozapine, synthesized in 1959, was soon considered different from other antipsychotics in both preclinical and clinical studies. At the preclinical level, clozapine did not induce cataplexy in rodents, while, at the clinical level, it was not associated with EPS, considered at that time an indirect marker of antipsychotic activity [14,15,16,17,18,19]. Quantification of D2R occupancy by PET and radioligand binding clearly demonstrated that the efficacy of antipsychotics could be achieved with D2R occupancy lower than the one responsible for the onset of EPS [8]. Elevated K_on_/K_off_ and consequent fast dissociation from the D2R have been proposed as an alternative hypothesis to explain atypicality [20].

Third-generation agents, such as aripiprazole, cariprazine, and brexpiprazole, have shown the common feature of occupying D2R as partial agonists as well as showing a multifaced receptor profile [12]. Cariprazine is demonstrated to be a D3R-preferred partial agonist and significantly more efficacious in schizophrenia with prevalent negative symptoms compared to D2R antagonists [21,22]. A more complex conceptualization of dopamine receptor partial agonists prompts from the observation that these drugs also work as biased ligands or functional selective molecules [23].

Apart from and beyond their action at dopaminergic and other central receptors, antipsychotic treatment has been demonstrated to modify brain expenditure energy metabolism and impact mitochondria function [24,25,26]. The relevance of regulating mitochondrial functions lies in the possible modulation of antioxidant intracellular pathways and other mechanisms involved in cell viability and survivance [26,27,28]. The potential modulation of mitochondria function and oxidative stress by antipsychotics is in line with a role for oxidative stress in schizophrenia and related psychosis [29,30,31,32].

Together with neurotransmitter dysfunction, neuroinflammatory processes, as well as mitochondrial alteration and oxidative stress reported in schizophrenia and animal models of psychosis, are believed to be involved in the pathophysiology of the disorder [26,28]. Inflammation has been associated with alterations in neurotransmission, neuronal signaling, synapse organization, and brain connectivity by increasing oxidative stress, inducing activation of microglia, and reducing gray matter volume [33,34,35,36,37]. Inflammatory processes and changes in oxidative pathways are highly relevant as they emerge during the early phases of life [28,38]. In line with the neurodevelopmental hypothesis of schizophrenia, exposure to pre- and postnatal stressors, including the cytokine storm and free radicals, could result in impairments in neuronal migration and neurite outgrowth [38,39,40].

The role of the gut microbiome in the pathogenesis of schizophrenia has been investigated, and a significant elevation of *Lactobacillus* group bacterial numbers was detected in psychotic patients compared to controls, which even correlated with symptom severity and treatment outcomes [41]. Gut dysbiosis could be implicated in the pathophysiology of schizophrenia by enhancing both intestinal and blood-brain barrier permeability, thus increasing the availability of circulating cytokines and regulating other brain signalings, such as the kynurenine and brain-derived neurotrophic factor (BDNF) pathways [42].

### Aims of the Study

Despite the recent evidence that supports the role of mitochondria in the action of antipsychotics, many aspects of this class of drugs on energy expenditure and oxidative stress remain unclear.

First, how and to what extent the effects of antipsychotics on mitochondria function are related to the efficacy of this class of drugs?

Second, considering the complexity of the receptor profile of antipsychotics and the occupancy of D2R as the main mechanism of their efficacy, is the antioxidant effect related to central modulation of dopamine or other receptor mechanisms?

Third, both antioxidant function and antipsychotic action have been demonstrated to affect the structure and dynamics of the synapse; which is the possible overlap, if any?

With these objectives in mind, we decided to use a comprehensive review of the available evidence on the role of antioxidants’ mechanisms in antipsychotic action with specific attention to mitochondrial dysfunction. We aimed to describe the potential differences between typical and atypical antipsychotics in modulating oxidative balance. We also focused on the interaction of dopamine and synaptic structure with oxidative stress and mitochondria, considering the effects exerted by antipsychotic compounds. Further, we systematically reviewed clinical trials conducted on psychotic patients exploring the efficacy and tolerability of antioxidant compounds as add-on treatments to antipsychotic medication. 

There is a growing interest in exploring new therapeutic strategies that might bypass the current deadlock in antipsychotic therapy, especially for those patients who do not respond or respond poorly to available antipsychotics and are considered treatment-resistant schizophrenia (TRS) patients.

## 2. Materials and Methods

### 2.1. Screening and Selection Process

**Figure 1:**
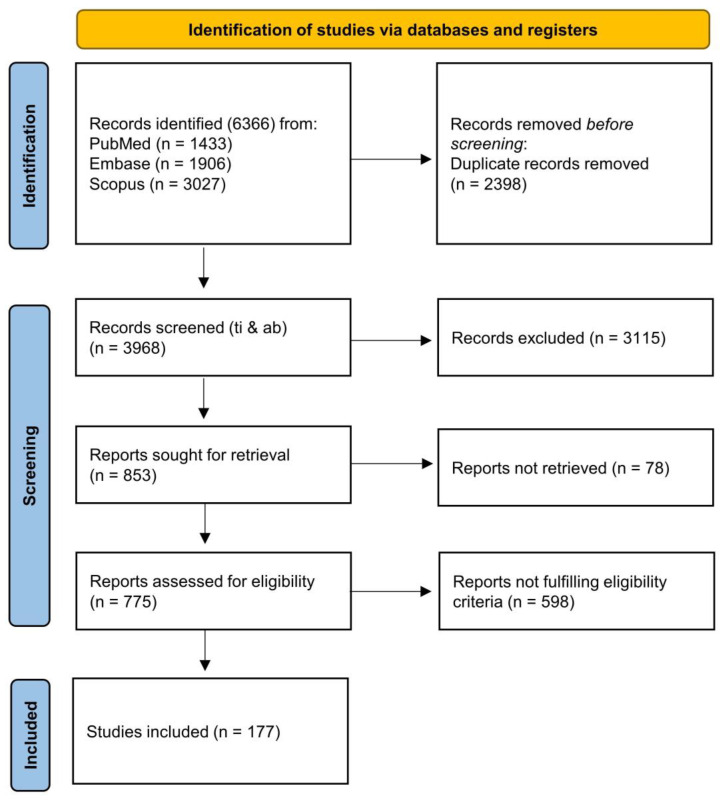
PRISMA flow diagram outlining the selection process through the different phases of the systematic review.

The present systematic review aims to provide an updated overview of the available evidence on the putative action of antipsychotics on mitochondrial function in the setting of schizophrenia and their potential antioxidant role. The selection process was conducted according to the Preferred Reporting Items for Systematic Reviews and Meta-Analyses (PRISMA) criteria [43] to identify eligible clinical and preclinical studies related to the topic. The articles from the primary search were selected independently by two authors (GDS and BM), and in the case of an inconsistent evaluation, the opinion of a third author (AdB) was sought. Other papers were searched manually by checking the reference lists of included articles as well as by matching multiple keywords emerging from the reading of the articles retrieved. The search returned a total of 6366 articles. After removing duplicates, 3968 references were identified. At the end of the screening process, 177 articles were included in the systematic review. The PRISMA flowchart is shown in Figure 1 to specify the individual steps of the selection procedure. The protocol of the present study was pre-registered on the International Platform of Registered Systematic Review and Meta-analysis Protocols (INPLASY202320106).

### 2.2. Search Strategy

EMBASE, Scopus, and Medline/PubMed databases were queried on 18 January 2023. The latest update was conducted on 15 February 2023. The search string adopted for the Medline/Pubmed database is reported as follows: (((((“schizophrenia”[Title/Abstract]) OR (“schizophrenia spectrum and other psychotic disorders”[MeSH Terms])) OR (“psychosis”[Title/Abstract])) OR (“psychotic disorder”[Title/Abstract])) OR (“antipsychotic”[Title/Abstract])) AND (((((“mitochondria”[Title/Abstract]) OR (mitochondrion[Title/Abstract])) OR (“mitochondria”[MeSH Terms])) OR (“free radical”[Title/Abstract])) OR (“antioxidant”[Title/Abstract])). The search strings for the other databases are reported in the Supplementary Materials.

### 2.3. Inclusion and Exclusion Criteria

We considered eligible English-language articles published in peer-reviewed journals, exploring potential oxidant/antioxidant effects of antipsychotics, the involvement of mitochondrial dysregulation in schizophrenia, and their implication in antipsychotic treatment. Clinical studies exploring the antioxidant/oxidant effects of antipsychotics in patients affected by psychotic disorders, such as chronic schizophrenia, FEP, and early psychosis, were included in the qualitative analysis. No time constraints were applied, and original clinical and preclinical research studies and reviews were included. Conference abstracts, commentaries, and letters to the editor were excluded. The PRISMA checklist has been provided in Supplementary Table S1. 

## 3. Molecular Abnormalities Driven by Inflammation and Oxidative Stress Relevant to Schizophrenia

### 3.1. Relevance of Inflammation in Schizophrenia

The possibility that inflammation may significantly impact the neurobiology of schizophrenia, contributing to alterations in neurotransmission, neuronal signaling, synapse organization, and brain connectivity, has emerged [33,34,35,36,44,45,46]. Consistent with multiple pieces of evidence, it has been reported that these alterations involve increased dopaminergic transmission in the striatal areas (putamen, caudate, and nucleus accumbens) and hippocampus [47,48,49,50]. In contrast, reduced dopaminergic transmission has been reported in the prefrontal cortex (PFC) with a subsequent clinical association of cognitive and behavioral disorders in schizophrenia [51]. The cooperation of the immune system and inflammatory response within the central nervous system (CNS) in several cognitive and behavioral functions has been proposed to be relevant in the association between schizophrenia and inflammation [52]. Dopamine may regulate inflammation through modulating migration, differentiation, and proliferation of immune cells (e.g., T cells, microglial cells, and peripheral monocytes) involved in cognitive function [45]. Alterations in dopamine levels associated with schizophrenia might influence the inflammatory/immune response and behavior, including spatial memory and learning, social behavior, and stress resilience [45]. On the other hand, the influence of inflammation on dopaminergic function has been demonstrated [53] with the increase of dopamine release and modulation of postsynaptic effects following inflammatory mechanisms in the CNS [54,55]. These findings suggest the putative implication of an active cross-talk between the dopaminergic and inflammatory processes in the pathophysiology of schizophrenia [45]. Another line of evidence linking dopamine and the inflammatory process via oxidative stress points to changes in subcortical dopaminergic activity as one of the circuits responsible for enhancing glutamatergic neurotransmission abnormalities in the substantia nigra [56]. The link between inflammation and glutamatergic transmission appears evident in models of maternal immune activation (MIA), where altered glutamate release and N-methyl-D-aspartate receptor (NMDAR) expression in the PFC and hippocampus [57] support the role of glutamate storm in the inflammatory process of neuroprogression [35]. The hypoglutamatergic hypothesis of schizophrenia could be integrated by the inflammatory contribution in the etiology of the disease, explaining the excessive synaptic pruning that occurs during late adolescence or early adulthood in schizophrenia [35,58,59]. Growing observations suggest that genetic and environmental risk factors for schizophrenia converge toward altered microglial function as a source of pro-inflammatory cytokines, nitric oxide (NO), and pro-oxidants during development in response to systemic and central oxidative and inflammatory events [60]. In this framework, targeting modulators of microglia activation could emerge as a potential early therapeutic intervention for schizophrenia treatment [61,62,63]. In summary, brain and systemic inflammation may contribute significantly to the development and progression of schizophrenia, placing oxidative stress and inflammatory mediators as potential biomarkers and targets for treating schizophrenia [64].

#### 3.1.1. Evidence from Preclinical Studies

Inflammatory-induced modifications at the level of postsynaptic density (PSD) scaffold proteins may affect downstream NMDAR signaling, leading to synaptic aberration and disruption of the dopamine–glutamate interaction [65]. Synaptic plasticity is possibly associated with abnormal learning, memory, and neural circuit maturation affected in schizophrenia [28,65]. It has been hypothesized that disrupted glutamatergic synaptic activity could impact the dysregulation of interneurons’ function in cortical circuits by disinhibiting control over dopamine release [66,67,68]. Integration of recent translational clinical evidence provides the hypothesis of synaptic overpruning in schizophrenia during a critical period of neurodevelopment due to an imbalance between excitatory glutamatergic pyramidal neurons and inhibitory gamma-aminobutyric acid (GABA)ergic interneurons, resulting in a localized glutamate storm that triggers excessive dendritic pruning and apoptosis [69,70]. It has been proposed that parvalbumin (PV^+^) interneurons are highly sensitive to NMDAR antagonism and vulnerable to oxidative stress, depending on antioxidant system regulation to neutralize the overproduction of mitochondrial reactive oxygen (ROS) species [71,72]. In experimental models, such redox dysregulation may induce abnormalities in neural connectivity and synchronization similar to those observed in patients with schizophrenia through hypoactive NMDARs, impairment of fast-spiking PV^+^ interneurons, and myelination deficits [71]. In this context, antipsychotic treatment counterbalancing alteration in the redox system could improve negative symptoms in patients with chronic schizophrenia [71]. Notably, dysfunction of GABAergic inhibitory interneurons induced a glutamate storm from excitatory glutamatergic cortical pyramidal neurons and a hyperdopaminergic subcortical state [35], corroborating the hypothesis that prenatal exposure to inflammation would result in an altered gene expression profile in the hippocampal structures and disruption in GABAergic, glutamatergic, and serotonergic neuronal circuits relevant to a model of inflammation-mediated psychosis [53,73,74]. Increased susceptibility to apoptotic mechanisms in schizophrenia with relevance to synaptic or dendritic loss [75,76] could be the consequence of both glutamate excitotoxicity and oxidative stress playing a prominent role, as well as the lack of neuroprotective factors (e.g., BDNF) [77,78]. A recent meta-analysis has indicated that reduced levels of the pre and postsynaptic proteins, such as synaptosome-associated protein of 25 kDa (SNAP-25), PSD-95, synapsin, and rab3A in the hippocampus and a reduction of synaptophysin in the hippocampus and frontal cortical regions are consistent with models involving a synaptic loss in schizophrenia [76,79,80,81,82,83]. Apoptotic loss of dendritic spines could be exacerbated by the inflammatory event of microglial activation from immune system responses (e.g., proteins of the MHC class I family and complement cascade protein C4) [59,84,85]. Microglia, astrocytes, and peripheral immune response cells are involved in the inflammatory processes and oxidative and nitrosative stress in schizophrenia [78]. Excessive reduction of dendritic spines and subsequent aberrant synaptic plasticity could be associated with brain disconnections and both negative and cognitive symptoms in schizophrenia [35]. An animal model of schizophrenia—using neonatal ventral hippocampal lesion (nVHL) in rats—evaluated the effect of the antipsychotic olanzapine, which was shown to correct the hyperlocomotion and impaired working memory of nVHL rats but did not improve their social behavior disorders [86]. This preclinical evidence suggests that in the PFC, olanzapine potentially reverses the structural plasticity of pyramidal cells, increasing the length of dendritic arches, spinogenesis, and the percentage of mature spines, as well as attenuates astrogliosis, oxidative stress, and apoptosis in the PFC [86]. MIA models involving cytokine network dysfunction culminate in microglia activation, impacting glial cell survival and contributing to morphological and functional changes in the brain [87,88]. Microglia activation leads to a mechanism by which both immunological and neuroplasticity-related factors via the elimination of weak or inactive synapses contribute to gray matter decline and altered functional connectivity reminiscent of schizophrenia [53,66,89]. Consistent with these findings, the activation of microglia leads to structural changes at the PSD and altered expression of genes involving NMDAR-dependent plasticity by decreasing the density of spines and synapses of glutamatergic cortical pyramidal neurons below the critical threshold for neural network functioning [90,91].

#### 3.1.2. Evidence from Clinical Studies

It has been postulated that increased pro-inflammatory mediators may alter brain development [92], consistent with the finding of multisystem biological dysregulation in individuals with schizophrenia [93]. Substantial evidence suggests that acute-phase immune signaling proteins are altered in first-episode psychosis (FEP) and high-risk psychosis subjects [94,95]. Furthermore, a meta-analytic study showed a relationship between oxidative stress markers and inflammatory cytokines and negative symptom severity in the FEP population naïve to antipsychotics, suggesting that early anti-inflammatory and antioxidant pharmacological interventions may have beneficial effects [96]. Schizophrenia is associated with aberrant redox regulation early in brain development [97]. Growing evidence reported that the antioxidant defense system could be closely associated with the therapeutic response to antipsychotics [98,99]. A clinical trial of FEP patients naïve to antipsychotics found higher total antioxidant status (TAS) levels than healthy controls. Baseline TAS levels were a predictor of symptom reduction after monotherapy of risperidone treatment, with a significant association between increased TAS levels and cognitive factor reduction, suggesting antioxidant protection related to risperidone treatment [100]. Sex differences in response to antipsychotic treatment and antioxidant defense system (ADS) have been reported. A clinical study showed that ADS enzymes were related to the antipsychotic treatment response in FEP patients. Specifically, female patients responded better to risperidone treatment than male patients, including changes in plasma glutathione peroxidase activities resulting as a favorable predictor of the response to risperidone treatment in female patients [101]. Oxidative stress plays a prominent role in antipsychotic-induced weight gain in schizophrenia; however, antioxidant enzymes might be involved in the weight gain caused by risperidone therapy. In this context, it has been reported that poor ASD may have a predictive value for weight gain in FEP patients after monotherapy of risperidone treatment [102]. A down-regulation of endogenous antioxidant and anti-inflammatory mechanisms has been reported in biological samples of schizophrenia patients, with a differential degree and progression from onset to full-blown illness [78]. In line with this evidence, the slow progression to FEP would represent a window of vulnerability to redox dysregulation and an opportunity for therapeutic interventions [77]. Clinical studies have shown in the early and late stages of chronic schizophrenia that serum mediators of oxidative stress and inflammation may act as biomarkers, reinforcing the value of early assessment of individuals at very high risk of developing psychosis and FEP subjects [96]. During the onset of psychosis, it has been reported that the antioxidant blood levels fall in schizophrenia patients with oxidative stress levels correlated with the severity of the symptoms. Redox imbalance could be a therapeutic target for preventive or adjuvant treatments, as well as biomarkers of disease progression, playing a role in the pathophysiology of emerging psychosis [103]. It has been reported that levels of thiobarbituric acid reactive substances (TBARS) and protein carbonyl content (PCC) are significantly higher in patients with schizophrenia than in healthy controls, with no differences in total reactive antioxidant potential (TRAP) and TNF-α levels between patients in the early and late stages of illness [96]. These findings support the hypothesis that the increased redox status and impaired antioxidative stress defense may accelerate synergistically neuronal degeneration in schizophrenia [96].

### 3.2. The Interaction between Oxidative Stress and Synaptic Plasticity 

Preclinical studies suggest that chronic oxidative stress increases inflammatory markers, inducible NO synthase (iNOS), and cyclooxygenase-2 (COX-2) in rats, triggering multiple intracellular changes, such as the high neuronal Ca^2+^ influx and the accumulation of ROS and reactive nitrogen species (RNS), disrupting synaptic transmission [104]. It has been proposed that oxidative stress-induced changes converge on the impairment of Na+/K+-ATPase (NKA) activity, representing the molecular basis of stress-related neuropsychiatric disorders, such as schizophrenia [104]. Although NKA plays a fundamental role in CNS functions [104] by modulating transmembrane ion gradients, it appears sensitive to the generation of NO and free radicals [105], resulting in membrane damage consistent with schizophrenia [106]. In the offspring of MIA models, the altered balance of Ca^2+^ levels and NO signaling in astroglial cells results in abnormalities of astrocyte structure and morphology with glutamatergic excitotoxicity and reduced survival of hippocampal neurons [107]. In addition, cytokines to acute neurodegeneration such as tumor necrosis factor (TNF)-α may increase glutamate-mediated cytotoxicity through two mechanisms: indirectly, by inhibiting glutamate transport on astrocytes, and directly, by triggering the surface expression of AMPARs and NMDARs while reducing inhibitory GABA-A receptors on neurons [108]. It has been suggested that the impact of TNF-α would alter the balance between excitation and inhibition with a relative increase in the excitatory/inhibitory synaptic ratio [108]. The molecular inflammatory scenario would also enhance activator protein 1 (AP-1) via the MAPK cascade, increasing the transcription of iNOS and COX-2 with ROS and RNS generation. Although neuronal death following glutamate excitotoxicity is also induced by activation of the AP-1 protein through non-transcriptional pathways, the deactivation of this peptide could have potential therapeutic applications directed at apoptotic and necrotic neuronal death [109,110,111]. Based on the hypothesis that a redox imbalance may explain the developmental alterations associated with schizophrenia, preclinical studies have investigated the injection of methylazoxymethanol acetate (MAM) in an animal model of altered neurodevelopment related to oxidative stress. Although the offspring of rats exposed to MAM treatment presented behavioral and neurochemical oxidative impairments in early adulthood consistent with those observed in schizophrenia, treatment with N-acetylcysteine (NAC) reversed the behavioral deficits through a recovery mechanism involving NO, suggesting a potential antipsychotic effect of NAC [112]. Combined evidence has shown that stress-induced changes in the glutamatergic system in the PFC appear to be biphasic. The rapid response to stress suggests increased synaptic transmission with increased numbers of excitatory synapses and working memory, whereas adaptive changes resulting from chronic long-term stress induce opposite effects [113]. This biphasic effect involved in stress-related disorders, such as schizophrenia, opens innovative paths for the development of novel therapeutic approaches. Similar effects have been observed in humans under chronic stress conditions and in animal models in which elevated pro-inflammatory cytokine signaling was associated with dendritic shrinkage and impaired neuronal functioning [113,114].

### 3.3. Antipsychotic Modulation Induced by Inflammation and Oxidative Stress in Schizophrenia

Alterations in dopaminergic transmission implicated in schizophrenia could influence the immune/inflammatory response and oxidative load, resulting in changes in behavioral functions in schizophrenia [45]. The role of tetrahydrobiopterin (BH4), an essential enzyme cofactor for tyrosine and dopamine synthesis regulated by inflammatory cytokines, could explain the close interaction between the dopaminergic system and the inflammatory state. In particular, cytokines and oxidative stress mediators may regulate the expression of GTP-cyclohydrolase 1 (GCH-1), an enzyme required for BH4 production, by promoting dopamine biosynthesis [115]. In fact, during inflammation, ROS and iNOS activity leads to a reduction in BH4, resulting in decreased dopamine synthesis [116,117]. In addition, vitamin C, folate, and other antioxidants would increase the bioavailability of BH4 at the endothelial level through chemical stabilization or scavenging of ROS, thus contributing to the maintenance of endothelial physiological homeostasis and possibly dopaminergic ones [116]. Low levels of vitamin C, which may partly affect the dopaminergic system, have been observed in patients with schizophrenia [118]. Although a clinical study reported that patients with schizophrenia had lower baseline levels of vitamin C, these did not correlate with the severity of disease symptoms [118]. Otherwise, higher levels of vitamin C at baseline were associated with a greater improvement in negative symptoms, suggesting a potential additional effect of vitamin C on antipsychotic treatment in schizophrenia [118]. The inflammatory/oxidative theory of the molecular pathophysiology of schizophrenia has raised relevant questions for the treatment of the disease and those forms that are poorly or unresponsive to canonical antipsychotic therapies such as TRS. The TRS condition may result in severe cognitive impairment and poor prognosis, which has also been associated with oxidative stress and deregulated inflammatory responses [119,120]. The involvement of the inflammatory system in influencing the response to antipsychotic action has been hypothesized. New therapeutic approaches targeting aberrant synaptic plasticity, glutamate storm, dendritic cell apoptosis, calcium channel dysfunction, and microglial activation have been explored, especially in the early stages of the disease. Preventing the loss of dendritic spines in high-risk individuals during the prodromal or transient phase of psychosis may be a potential target in the treatment of schizophrenia [70,121,122,123]. Clozapine, the only antipsychotic drug approved for TRS, has been shown to increase PSD proteins and promote the formation of dendritic spines [124,125]. However, clozapine showed inhibitory effects on dopaminergic activity in the midbrain [126], mediated by its action on the glycine binding site located on the NMDAR [127], mitigating the symptom dimension in schizophrenia [126]. Alpha lipoic acid (ALA) has been held responsible for reversing NMDAR hypofunction, as well as blocking dopamine receptors and increasing neurotrophic factors with an antioxidant effect [128]. Its potential use in TRS has been assessed in a randomized clinical trial (RCT) study that found a significantly greater improvement in the test group compared with the control in the Scale for the Assessment of Negative Symptoms (SANS) scores. ALA supplementation improved psychopathology and decreased oxidative stress in patients with TRS [128]. In contrast, another RCT in patients with schizophrenia under supplementation with ALA found no significant improvement in body mass index, cognition, psychopathology, adverse effects of antipsychotics or oxidative stress, and inflammation in the experimental group compared to placebo. Significant decreases in red blood cells, white blood cells, and platelets were found in the entire ALA-treated group [129]. It has been reported that antipsychotic drugs can reduce inflammatory and oxidative effects by acting on the dopaminergic system [45,130,131]. On the other hand, inflammation-induced susceptibility and oxidative stress could also explain the glutamatergic hypothesis of schizophrenia through the developmental abnormalities described in the “two-hit model,” in which genetic factors combined with environmental insults contribute to the development of the disease [132]. Anti-inflammatory and antioxidant therapy in add-on to antipsychotics is a growing field of interest that needs further research [132,133,134].

## 4. General Appraisal of Antioxidant Pathway and Mitochondria Dysfunction in Schizophrenia

Several alterations in mitochondrial function have been associated with the pathophysiology of schizophrenia, including impairments in mitochondrial complex one (MCI) and glutathione pathway, which may represent a biological link to dysfunction of dopaminergic and glutamatergic signaling and oxidative stress [26]. 

Although one postmortem study failed to show significant differences in MCI function between patients with schizophrenia and controls [135], lower MCI activity was detected in the prefrontal [136] and temporal [137] cortices as well as basal ganglia [137] of patients compared to controls. In addition, reduced expression of genes encoding for MCI subunits has been described in postmortem studies conducted on patients with psychotic disorders [138]. Specifically, mRNA levels of Ubiquinone Oxidoreductase Core Subunit V1 (NDUFV1), NDUFA8, NDUFB9, NDUFS4, NDUFS7, and NDUFV2 were decreased in the frontal cortex of patients with schizophrenia compared with controls [26,138]. In contrast, the mRNA expression of NDUFB2, NDUFB5, and NDUFS4 was decreased in the hippocampus [139] and the mRNAs of NDUFV1, NDUFV2, and NADUFS1 in the striatum of patients with schizophrenia [140].

The glutathione system exerts a key survival antioxidant function in brain mitochondria by scavenging free radicals and protecting intracellular membranes against oxidative damage [141,142]. Clinical and preclinical findings have linked glutathione to schizophrenia pathophysiology [143]. The activity of fast-spiking parvalbumin interneurons, whose implication in the pathogenesis of schizophrenia has been suggested, as well as dendrite morphology in the CA1 region of the hippocampus, were altered in mice with a genetically compromised glutathione synthesis [144,145,146]. The glutathione system was also found to protect neuronal cells from dopamine toxicity and regulate mitochondrial functions. In fact, a significant decrease in the number of neuronal processes was reported in cultured cortical neurons incubated with dopamine in a state of low glutathione concentrations [147]. A correct ratio of reduced/oxidized glutathione was found essential to regulate mitochondrial dynamics in neurons by affecting mitofusin (MFN) and optic atrophy 1 (OPA1) activity in the fusion pathway [148]. In addition, glutathione depletion was associated with disruptions in myelin formation and maturation [149,150,151,152]. Overall, these alterations are consistent with the modification observed in dendritic spines reported in schizophrenia [1] and possibly related to abnormalities in synaptic and circuit connectivity, representing the neurobiological underpinning of psychotic symptoms and a possible target for antipsychotic treatment [6]. In preclinical models of schizophrenia, treatments with antioxidant agents, such as NAC, that increase glutathione levels were associated with improvement in cognitive and social behaviors [153,154,155], an elevation in the number of parvalbumin cells [156,157], prevention of deficits in evoked-related potentials measured by electroencephalography (EEG) electrodes and in the prepulse inhibition of the acoustic startle response [157], an increase in mitochondria numbers and myelin-related mRNA expression [155], with higher benefits if the administration occurred at an early stage of neurodevelopment. Alterations in glutathione levels have been studied in vivo by magnetic resonance spectroscopy (MRS) conducted on patients with psychotic manifestations [158]. Lower levels of glutathione and decreased activity of glutathione peroxidase were found in patients affected by schizophrenia, especially in those without medications, compared to controls [159]. A meta-analysis of MRS studies found a significant reduction of glutathione levels in the anterior cingulate cortex (ACC) of schizophrenia patients [160]. In FEP patients, lower levels of glutamate and glutathione were detected in the ACC, while N-acetylaspartate was found to be decreased in the orbitofrontal region [161]. Both glutathione and N-acetylaspartate levels were reduced in the thalamus [161]. In a study including FEP patients, higher levels of glutathione were found in the dorsal ACC and considered indicative of a more favorable prognosis [162]. Specifically, glutathione levels were associated with inhibitory activity exerted by GABAergic neurons within the dorsal ACC and the anterior insular cortex [162]. In this regard, the antioxidant state could be considered a mechanism to reverse the glutamate-mediated dysconnectivity and counterbalance the hyperglutamatergic state due to the disinhibition of GABA neurons [162]. Curiously, treatment-resistant patients showed elevated glutamate concentration in the ACC compared to treatment-responsive individuals [163,164], whereas no difference was detected in glutathione levels between groups based on different responsiveness to treatments [165].

Other findings related to altered neuronal mitochondria have been variously addressed to the pathophysiology of schizophrenia [166].

Reduced density and the total number of neuronal mitochondria were found in the caudate and putamen of postmortem brains in untreated patients with schizophrenia compared with healthy controls and drug-treated subjects, suggesting that antipsychotic treatment may reverse this alteration [167,168]. Other significant reductions in mitochondria number and density were detected in oligodendrocytes [169,170], astrocytes [171], and microglia cells [169] of psychotic patients within the hippocampus [171], PFC [169], and white matter [170].

In addition, several single nucleotide polymorphisms (SNPs) detected in the mitochondrial deoxyribonucleic acid (mtDNA) have been associated with schizophrenia, including the gene encoding NADH dehydrogenase of the MCI [172], the gene encoding ATP synthase subunit six [173], and the gene encoding a tRNA (Leu(UUR)) [174].

**Figure 2:**
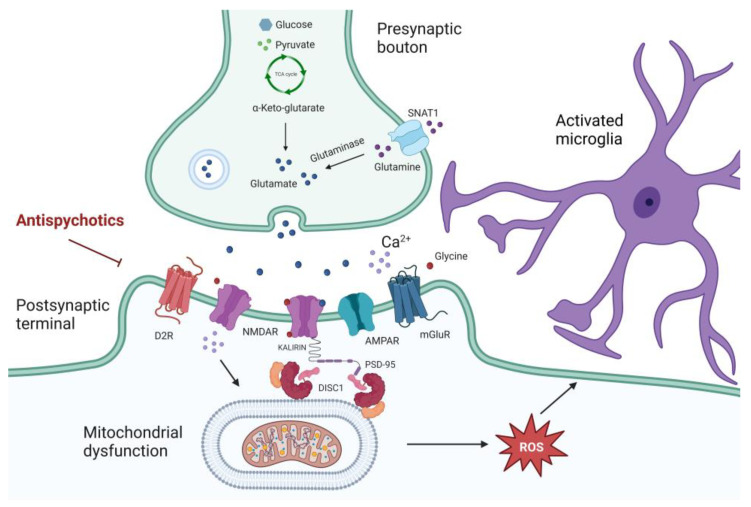
Mitochondrial dysfunction in schizophrenia can result in abnormal free radicals production and oxidative stress, which can be variously modulated by antipsychotic treatment. DISC1, a component of PSD with a putative role in mitochondrial fission and fusion, is also involved in schizophrenia pathophysiology. AMPAR = α-amino-3-hydroxy-5-methyl-4-isoxazolepropionic acid receptor; D2R = dopamine D2 receptor; Ca^2+^ = calcium ion; DISC1 = disrupted-in-schizophrenia 1; mGluR = metabotropic glutamate receptor; NMDAR = N-Methyl-D-aspartic acid receptor; PSD-95 = postsynaptic density protein 95; SNAT1 = sodium-coupled neutral amino acid transporter 1. Created with BioRender.com; last accessed on 21 February 2023.

Mitochondrial fission and fusion are essential processes involved in neurodevelopment and are regulated by different proteins, including MFN and OPA1 [166]. Deletion of *MFN2* or *OPA1* genes, when present globally in the CNS, is lethal in mice [175,176]. Otherwise, cerebellar-specific deletion of MFN2 in mice was characterized by impairment in fusion and dendritic localization of mitochondria, together with altered dendritic spine development and free radicals’ production [177]. In rodent cortical primary neurons, the downregulation of OPA1 was associated with an increase in fragmented mitochondria and a reduced expression of mitochondrial respiratory complexes, along with a decrease in the number of synapsis and protein levels of postsynaptic structural components [178]. A reduction of OPA1 protein levels, as well as structural perturbations in the mitochondrial network, were observed in the PFC of patients diagnosed with schizophrenia when compared to healthy controls [179]. SNPs in the gene encoding G72, the activator of D-amino acid oxidase (DAO), were found to be associated with major psychiatric disorders, including schizophrenia [180]. One splicing isoform of *G72* encodes for a mitochondrial protein endowed with the capability to enhance mitochondrial fragmentation and dendritic arborization [181]. Three common *disrupted in schizophrenia 1 (DISC1)* gene variants as well as several rare and ultrarare variants, have been associated with schizophrenia [1]. Beyond the well-established role in the PSD composition, splicing isoforms of *DISC1* have also been localized in cell mitochondria [182]. Overexpression of DISC1 has been linked to the formation of mitochondrial ring structures, indicating potential involvement in mitochondrial fusion and fission [182], probably by the direct interaction with MFN1 and MFN2, the outer mitochondrial membrane GTPase proteins Miro1 and Miro2, and the TRAK1 and TRAK2 mitochondrial trafficking adaptors [183]. A graphical representation of mitochondrial dysfunction and DISC1 impairments involved in schizophrenia pathophysiology has been provided in Figure 2.

Overall, these data suggest a strong interplay between schizophrenia and disturbances in cell oxidative mechanisms, especially considering the role exerted by the MCI and glutathione system in neuronal mitochondria.

## 5. Antipsychotics: An Oxidant or Antioxidant Effect in Schizophrenia?

Antipsychotics have been seen to modulate oxidative processes, thereby influencing the course and progression of schizophrenia, with differences between first- and second-generation compounds as outlined in Table 1 [27].

### 5.1. Effects of Typical Antipsychotics

Haloperidol, a first-generation antipsychotic with a strong D2R antagonism, has antioxidant and neuroprotective properties as well as oxidant and neurotoxic effects. Apart from being a strong antagonist at the D2R, haloperidol has been recognized to tackle sigma1 receptors [184], exerting a possible neuroprotective effect [185]. A recent study has investigated the potential capability of haloperidol to mitigate processes of oxytosis/ferroptosis, an iron-dependent non-apoptotic form of regulated cell death, in sigma1 receptor knockdown mouse hippocampal HT22 cells [185]. The results have shown that haloperidol could exert neuroprotective effects through mechanisms independent of the sigma1 receptors [186]. By the application of fluorescent probes, haloperidol was localized to endosomes and lysosomes and was shown to reduce the accumulation of Fe^2+^ and prevent the phenomena of cell death [186].

The putative neuroprotective action of haloperidol should be critically evaluated considering the findings indicating, oppositely, that this drug may have relevant neurotoxic and genotoxic effects on neuronal cells [27]. Several mechanisms have been proposed to explain the detrimental effect of haloperidol and other first-generation antipsychotics on cell survival and neuronal oxidative pathways [187].

In the mouse frontal cortex, haloperidol was seen to decrease BDNF, glutathione, and B-cell lymphoma-extra large (Bcl-xL) levels [188]. Of interest, glutathione exerts a significant antioxidant role in cell mitochondria, and, on the other hand, Bcl-xl localizes at different intracellular organelles, including endoplasmic reticulum, Golgi apparatus, peroxisomes, and mitochondria [141,189]. Neuronal mitochondria should be considered a relevant site for possible oxidant and neurotoxic consequences. In this regard, haloperidol administration resulted in a six-fold increase in the levels of oxygen free radicals generated by mitochondria, together with a reduction in glutathione and a concomitant elevation in intracellular Ca^2+^ flux, as evaluated in rat primary cortical neurons and the mouse hippocampal cell line HT22 [190]. The administration of cystamine, an antioxidant and anti-apoptotic compound, effectively prevented a haloperidol-induced reduction in BDNF, glutathione, and Bcl-xl protein levels in mice via the Tropomyosin receptor kinase B (TrkB)/Akt pathway [188]. Haloperidol administration in mice was observed to induce a decrease in glutathione concentration in the cortex, striatum, and cerebellum, which was reverted by the simultaneous treatment with cinnarizine [191]. Beyond the amelioration of the haloperidol-induced brain oxidative stress, cinnarizine, a blocker of voltage-gated Ca^2+^ currents, effectively inhibited the mitochondrial permeability transition, involved in apoptosis and cell death [192]. The administration of haloperidol was associated with an enhancement of the NF-κB transcription factor [193], inhibition of the Akt pathway [194], and elevation in the proapoptotic protein caspase-3 levels [194]. Further, a potentially neurotoxic pyridinium metabolite was identified in the brain tissue of rats treated with haloperidol [195]. The study of haloperidol toxicity in human peripheral blood lymphocytes showed that the drug was able to induce significant oxidative stress by reducing glutathione levels, increasing lipid peroxidation, and exerting genotoxic effects [196]. The adjunctive treatment with carvacrol effectively countered the haloperidol-induced genetic damage, probably via the scavenging of free radicals and increased glutathione concentration [196].

A preclinical study conducted on rats treated with chlorpromazine, a first-generation drug, showed that this compound has neuroprotective effects through inhibition of the mitochondrial apoptotic pathway. Specifically, results correlate chlorpromazine use with decreased expression of cleaved caspase-3, cytochrome c, and Bax and increased expression of Bcl-2 and tissue factor (TF) [197]. In rat brains, the long-term administration of chlorpromazine significantly reduced the activity of superoxide dismutase (SOD), a cytoplasmatic and mitochondrial enzyme involved in the scavenging of the superoxide (O^−2^) radical [198].

Overall, haloperidol exerts notable neurotoxic effects at different doses via several molecular mechanisms that lead to neuronal death and oxidant damage. A similar effect was observed with other first-generation antipsychotics, whereas second-generation compounds induced only a few neurotoxic damages and occurred mainly at high doses [187]. Otherwise, contrasting evidence on the inflammatory/anti-inflammatory effect of haloperidol is reported in the literature. Haloperidol, as well as risperidone, clozapine, and quetiapine, were all effective in raising anti-inflammatory (interleukin [IL]-4 and IL-10) cytokine levels in peripheral mononuclear blood cell cultures extracted from twelve patients with FEP [199]. In human neuroblastoma SK-N-SH cells, treatment with haloperidol was linked to an increase in caspase-3 activity and cell death, whereas second-generation antipsychotics, such as risperidone and paliperidone, decreased caspase-3 activity and did not affect cell viability or cell death [200]. In astrocyte-like cells, while incubation with risperidone significantly increased glutamate uptake, glutamine synthase activity, and glutathione content, high doses of haloperidol increased ROS production [201]. The administration of haloperidol but not risperidone induced apoptotic injury in cultured cortical neurons, together with reduced Akt phosphorylation and an increase in caspase-3 activity [194]. Interestingly, the neurotoxicity related to haloperidol was partially reduced by treatment with BDNF, the D2R agonist bromocriptine, and ketanserin, a 5HT_2A_R antagonist [194].

### 5.2. Effects of Atypical Antipsychotics

Dysfunctions in the antioxidant and anti-inflammatory systems, unsaturated fatty acid metabolism, and alterations in sulfation-related mechanisms have been reported in first-episode, drug-naive patients with schizophrenia through a metabolomic analysis conducted on compounds detected using liquid chromatography-mass spectrometry [202]. Specifically, a biomarker panel including alpha-dimorphecolic, PC(16:0/18:1(11Z)), 1-methylnicotinamide, PE(20:2(11Z,14Z)/18:2(9Z,12Z), sulfate, and L-tryptophan was shown to differentiate schizophrenia patients from healthy controls with an area under the ROC curves (AUC) of 0.972 [202]. On the other hand, differences not based on the disease group and induced by antipsychotic treatment also were shown [202]. A biomarker panel including C16 sphinganine, gamma-linolenic acid, linoleic acid, C(16:0/18:1(11Z)), PE(20:2(11Z,14Z)/18:2(9Z,12Z)), and sulfate, was able to discriminate between patients before and after medication with an AUC of 0.905 [202]. Of interest, all patients enrolled in the study were treated with second-generation antipsychotics, such as risperidone and olanzapine, for 4–6 weeks at an optimal dose [202]. In another study conducted on 179 psychotic patients, a three-month treatment with risperidone and olanzapine was associated with an improvement in oxidative balance, as proved by an increase in blood glutathione, vitamin E, and vitamin C levels parallelized by a decrease in malondialdehyde concentration, a lipid peroxidation marker [31].

Risperidone treatment significantly decreased SOD blood levels in forty-one schizophrenia patients [203]. The reduction in SOD levels positively correlated with a decrease in the Positive and Negative Syndrome Scale (PANSS) negative subscale [203]. This evidence is relevant since, in chronic schizophrenia, SOD expression was found to increase, probably due to oxidative phenomena taking place in patients [27].

Divergent evidence on olanzapine-mediated regulation of the balance between oxidant and antioxidant factors has been reported. Olanzapine can induce increased oxidative stress and mitochondrial dysfunction, triggering cytotoxic mechanisms and neural death. A possible antidote to these events is represented by NAC use [204]. On the other hand, in PC12 cells exposed to hydrogen peroxide, pretreatment with olanzapine was effective in attenuating the decrease in cell viability and SOD-1 mRNA levels as well as increasing SOD enzymatic activity [205].

In a cross-sectional study conducted on two groups of fifty patients affected by schizophrenia, chronic olanzapine administration was associated with lower levels of TBARS, indicators of altered lipid peroxidation, and higher antioxidant values than treatment with haloperidol [29]. Olanzapine, different from clozapine, chlorpromazine, and haloperidol, was seen to inhibit the activity of the human D-aspartate oxidase (DASPO) as measured by in vitro enzymatic assay [206]. Similarly, risperidone was shown to exert a partial uncompetitive inhibition effect on human DAO and exhibited a protective effect from D-amino acid-induced cell death, as assessed by in vitro experiments [207]. Its relevance for schizophrenia treatment lies not only in increasing D-aspartate and D-serine levels, which can modulate NMDAR activity, but also in reducing oxidative reactions and free radical production [208]. In fact, DASPO and DAO are peroxisomal enzymes involved in the oxidative deamination of acidic D-amino acids, including D-aspartate and D-glutamate, and D-serine, respectively, resulting in α-keto acid, ammonium, and hydrogen peroxide (H_2_O_2_) production [208]. The administration of a DAO inhibitor, such as 5-chloro-benzo[d]isoxazol-3-ol (CBIO), in a preclinical model of schizophrenia enhanced D-serine efficacy in mice. In contrast, the D-amino acid alone was not effective in attenuating the pre-pulse inhibition deficit induced by MK-801 treatment, probably due to lower oxidative stress [209,210].

Among other antipsychotic drugs, clozapine and olanzapine seem to exert the most antioxidant properties. The neuroprotective and antioxidant features of six different antipsychotics were evaluated via in vitro models [211]. Clozapine and olanzapine showed to be more effective in scavenging superoxide anion on the respiratory burst and stabilizing free radicals than risperidone and ziprasidone, probably due to the amino group part of their chemical structure. In contrast, quetiapine and haloperidol completely lacked antioxidant effects [211]. Analogously, clozapine significantly enhanced SOD levels and reduced lipid peroxidation in comparison to perphenazine and risperidone [30]. Clozapine also increased peripheral glutathione, whose levels negatively correlated with the PANSS negative subscale [30]. In addition, ziprasidone and haloperidol but not clozapine significantly raised TBARS levels in the plasma of healthy subjects after 24 h of incubation, indicating a status of increased lipid peroxidation [212].

The oxidant/antioxidant action of aripiprazole, a third-generation antipsychotic characterized by a partial agonism on D2R and D3R and antagonism at the 5HT_2A_ receptor, has also been extensively studied in clinical and preclinical models [27]. Contrary to the oxidant effect of risperidone, ziprasidone, or haloperidol, aripiprazole had no effect on lipid peroxidation, assessed as TBARS levels in the plasma of healthy volunteers [213]. Of interest, a dose-related effect was detected, with higher doses of aripiprazole associated with a more pronounced antioxidant action [213]. The impact of aripiprazole on cell proliferation and neuronal differentiation was evaluated in the dentate gyrus of adolescent mice by immunohistochemistry [214]. Aripiprazole was effective in enhancing cell proliferation and maturation and complexity of neuroblast dendrites, along with decreased lipid peroxidation and increased SOD2 levels [214].

Xanomeline, a recent antipsychotic with agonism at the muscarinic M1 and M4 receptors (M1/4R), was tested on rat cortical neurons and showed to be effective in protecting the cells from oxygen-glucose deprivation (OGD)-induced injury and cell apoptosis, inhibiting ROS production induced by OGD, and preventing p-Akt reduction [215].

Altogether, these findings suggest different antioxidant/oxidant potentials between first- and second-generation compounds, with possibly better benefits obtained from clozapine and olanzapine administration.

**Table 1:** Oxidant and antioxidant effects of first- and second-generation antipsychotics.

Antipsychotics	Study Design	Model	Main Outcomes	References
Haloperidol	Preclinical study	S1R-knockdown mouse hippocampal HT22 cells	HAL could exert neuroprotective effects through mechanisms independent of sigma1 receptors. By the application of fluorescent probes, HAL was localized to endosomes and lysosomes and was shown to reduce the accumulation of Fe^2+^ and prevent the phenomena of cell death.	Hirata et al., 2022 [186]
	In vivo preclinical study	Adult male CD1 mice	In the mouse frontal cortex, HAL was seen to decrease BDNF, GSH, and Bcl-xL levels.	Pillai et al., 2008, [188]
	Preclinical study	Rat primary cortical neurons and mouse hippocampal cells line HT22	HAL resulted in a six-fold increase in the levels of ROS generated by mitochondria, together with a reduction in GSH and a concomitant elevation in intracellular Ca2+ flux in rat primary cortical neurons and mouse hippocampal cells.	Sagara 1998 [190]
	Preclinical study	Mice	HAL induced a decrease in GSH concentration in the cortex, striatum, and cerebellum, which was reverted by the treatment with cinnarizine.	Abdel-Salam et al., 2012 [191]
	Preclinical study	Human peripheral blood lymphocytes	HAL induced increased oxidative stress, reduced GSH levels, increased lipid peroxidation, and exerted genotoxic effects in human lymphocytes.	Zamani et al., 2022 [196]
Haloperidol and risperidone	In vitro preclinical study	Cultures of cortical neurons of rats at E18	HAL reduced Akt phosphorylation levels and caspase-3 activation; instead, BDNF could reverse these HAL-induced effects. Furthermore, only HAL, not RIS, could induce caspase-dependent apoptosis.	Ukai et al., 2004 [194]
	In vitro comparative study	Cultures of astrocyte-like cells (C6 cell line)	RIS increased glutamate uptake, glutamine synthase activity, and GSH content, and high doses of HAL increased ROS.	Quincozes-Santos et al., [201]
Ziprasidone, clozapine, and haloperidol	In vitro preclinical study	Human blood	ZIP and HAL but not CLO raised TBARS levels in the plasma of healthy subjects after 24 h of incubation, indicating a status of increased lipid peroxidation.	Dietrich-Muszalska et al., 2013 [212]
Risperidone, ziprasidone, haloperidol, aripiprazole	In vitro preclinical study	Human blood	Contrary to the oxidant effect of RIS, ZIP, or HAL, APZ had no effect on lipid peroxidation, assessed as TBARS levels in the plasma of healthy volunteers.	Dietrich-Muszalska et al., 2018 [213]
Haloperidol, risperidone, and paliperidone	In vitro comparative study	Human neuroblastoma SK-N-SH cells	HAL was linked to an increase in caspase-3 activity and cell death RIS and PP decreased caspase-3 activity and did not affect cell viability or cell death.	Gassó et al., 2012 [200]
Aripiprazole	Preclinical study	Adolescent male ICR mice	APZ was effective in enhancing cell proliferation and maturation and complexity of neuroblast dendrites in the dentate gyrus, along with decreased lipid peroxidation and increased SOD2 levels.	Chen et al., 2013 [214]
Chlorpromazine	Preclinical study	Rats	CPZ has neuroprotective effects through mitochondrial apoptotic pathway inhibition. Moreover, CPZ decreased the expression of cleaved caspase-3, cytochrome c, and Bax and increased the expression of Bcl-2 and TF.	Wu et al., 2016 [197]
	Preclinical study	Rats	The long-term administration of CPZ reduced the activity of SOD.	Abdalla et al., 1994 [198]
Risperidone	Clinical trial	40 patients with SCZ	RIS decreased the initially high blood levels of SOD observed in patients with SCZ.	Zhang et al., 2003 [203]
	In vitro preclinical study	Rat C6 cells were transfected with a plasmid encoding mouse DAO	RIS exerted a partial uncompetitive inhibition effect on human DAO and exhibited a protective effect from D-amino acid-induced cell death.	Abou El-Magd et al., 2010 [207]
Olanzapine	In vitro preclinical study	mHypoA-59 hypothalamic neuronal cell line	OLA could increase ROS and mitochondrial dysfunction, triggering cytotoxic mechanisms and neural death. A possible antidote to these events is represented by NAC use.	Boz et al., 2020 [204]
	Preclinical study	PC12 cells	In PC12 cells exposed to hydrogen peroxide, pretreatment with OLA attenuated the decrease of SOD-1 mRNA levels, while SOD enzymatic activity increased.	Wei et al., 2023 [205]
Haloperidol, risperidone, clozapine, and quetiapine	Preclinical study	Cultures of peripheral mononuclear blood cells extracted from 12 patients with FEP	HAL, as well as RIS, CLO, and QTP, were all effective in raising anti-inflammatory cytokine (IL-4 and IL-10) levels.	Al-Amin et al., 2013 [199]
Haloperidol and olanzapine	Cross-sectional study	50 patients with SCZ	Chronic OLA administration was associated with lower TBARS levels, which are indicators of altered lipid peroxidation, and higher antioxidant values than treatment with HAL.	Singh et al., 2008 [29]
Risperidone and olanzapine	Longitudinal study	179 psychotic patients (93 of them completed the study)	Three-month treatment with RIS and OLA was associated with an increase in blood GSH, α-tocopherol, and ascorbic acid levels parallelized by a decrease in malondialdehyde concentration, a lipid peroxidation marker.	Zerin Khan et al., 2018 [31]
Clozapine, olanzapine, haloperidol, quetiapine, risperidone and ziprasidone	In vitro preclinical study	Human neutrophils	CLO and OLA showed to be more effective in scavenging superoxide anion on the respiratory burst and stabilizing free radicals than RIS and ZIP, probably due to the amino group part of their chemical structure, whereas QTP and HAL completely lacked antioxidant effects	Brinholi et al., 2016 [211]
Olanzapine and clozapine	Preclinical study	KO male mice for the Ddo	OLA, different from CLO, CPZ, and HAL, was seen to inhibit the activity of the human DASPO.	Sacchi et al., 2017 [206]
Perphenazine, clozapine, and risperidone	Observational study	100 patients with SCZ	CLO showed to enhance SOD levels and reduce lipid peroxidation in comparison to PPZ and RIS.	Hendouei et al., 2018 [30]
Xanomeline	Preclinical study	Rat cortical neurons	Xanomeline was effective in protecting cells from OGD-induced injury and cell apoptosis, inhibiting ROS production induced by OGD, and preventing p-AKT reduction	Xin et al., 2020 [215]

Akt = serine/threonine kinase; APZ = aripiprazole; Bcl-Xl = B-cell lymphoma-extra large; BDNF = brain-derived neurotrophic factor; Ca^2+^ = calcium ion; CD1 = cluster of differentiation 1; CLO= clozapine; CPZ = chlorpromazine; DAO = D-amino acid oxidase; DASPO= D-aspartate oxidase; Ddo = D-aspartate oxidase; E18 = embryonic day 18; Fe^2+^= ferrous ion; FEP = first episode psychosis; GSH = glutathione; HAL = haloperidol; IL = interleukin; KO = knockout; mRNA = messenger ribonucleic acid; NAC = N-acetylcysteine; OGD = oxygen-glucose deprivation; OLA = olanzapine; PP = paliperidone; PPZ = perphenazine; QTP = quetiapine; RIS = risperidone; ROS = reactive oxygen species; S1R = sigma-1 receptor; SCZ = schizophrenia; SOD = superoxide dismutase; TBARS = thiobarbituric acid reactive substances; TF = tissue factor; ZIP = ziprasidone.

## 6. Oxidative System, Dopamine, and Mitochondrial Dysfunction: An Interplay Possibly Relevant for Schizophrenia and Antipsychotic Treatment

Mitochondrial dysfunction and ROS production may play a role in the pathophysiology of multiple psychiatric disorders, such as bipolar disorder and schizophrenia, through neurotransmitter mechanisms partly yet to be elucidated [172,216,217]. However, increased dopamine release at the subcortical level and the dysregulation of signaling are at the core of the pathophysiology of schizophrenia and the main target of the action of antipsychotics via D2R occupancy [12]. Therefore, it is conceivable that any effort to understand how antipsychotics may affect energy expenditure in the brain should consider the associaton with dopamine or, in general, catecholamines. The dopamine and catecholamine signaling may have evolved as possible regulators of energy metabolism and mitochondrial respiration pathways, sharing a common set of biosynthetic and metabolic enzymes. It has been suggested that this process may be the result of “retrofitting enzymes” during evolution [218].

Multiple pieces of evidence have linked the dopamine pathway to mitochondrial function in terms of oxidative stress.

Specifically, it was demonstrated that chronic exposure to amphetamines, which are known to enhance dopamine levels via dopamine transporter (DAT) blockage, increased superoxide production in submitochondrial particles in the PFC and hippocampus of rats [219]. Moreover, mitochondria are vulnerable to high concentrations of dopamine [216]. Dopamine altered the mitochondrial membrane potential and reversibly inhibited the activity of the mitochondrial complex [216,220]. In this context, antipsychotics may act on dopaminergic neurotransmission and prevent mitochondrial oxidative stress [219].

Evidence showed that dopamine, when added to cultured cortical neurons, contributed to decreases in GSH levels, reduced the number of neuronal spines, and increased dendritic degeneration and ROS production [147,221].

A postmortem study investigated the presence of metabolic abnormalities related to cytochrome c oxidase (COX) dysfunction in the substantia nigra and ventral tegmental area of patients with schizophrenia and a reduced protein expression of COX subunits II and IV was detected in these patients compared to non-psychiatric controls [222]. Otherwise, significant changes in COX were not detected in rats chronically treated with antipsychotic drugs [222].

Several studies explored the relationship between SOD activity and tardive dyskinesia, a movement disorder associated with chronic antipsychotic therapy and probably due to D2R signaling enhancement [223]. Reduced blood SOD levels and activity, as well as SNPs in the *SOD* gene, have been found in patients affected by tardive dyskinesia, indicative of dysfunction of protective mechanisms from free radicals’ production in these patients [224]. Of interest, second-generation antipsychotics, such as olanzapine and clozapine, which are less likely to induce tardive dyskinesia, were found to increase SOD expression, as discussed above [30,205].

The l-butionin-(S, R)-sulfoximine (BSO), an inhibitor of GSH synthesis, and GBR 12909, a dopamine reuptake inhibitor, administered in rats during early postnatal development, affected ROS levels, lipid peroxidation, and the activity of some antioxidant enzymes, such as SOD, catalase, glutathione peroxidase, and reactive glutathione disulfide [225]. These changes were found in peripheral tissues as well as in the PFC, hippocampus, and striatum of rats [225]. Specifically, in the hippocampus, GBR 12909 was associated with reduced SOD and glutathione peroxidase activity, whereas in the PFC and striatum, SOD activity was enhanced by both GBR 12909 and BSO administration [225]. In addition, it was observed that the combined treatment of the two substances (the glutathione synthesis inhibitor and the dopamine reuptake inhibitor) resulted in a significant decrease in lipid peroxidation levels and the absence of ROS changes in the PFC and hippocampus of rats [225]. All these early events may trigger the appearance of behavioral abnormalities and the development of schizophrenia in adulthood [225].

In another study, BSO was shown to reduce the levels of BDNF mRNA and protein in both the PFC and hippocampus of rats [226]. The combination of BSO with a dopamine reuptake inhibitor reduced BDNF mRNA and proteins only in the PFC, while in the hippocampus, only the protein levels of BDNF were decreased [226]. It was also observed that rats treated with BSO alone and those with BSO in combination with the dopamine reuptake inhibitor showed behavioral abnormalities similar to the schizophrenia phenotype [226]. However, only in combination-treated rats did amphetamine administration exacerbate positive symptoms in adulthood [226]. This highlights that schizophrenia may be triggered by redox abnormalities associated with disruption of dopaminergic transmission, with inhibition of dopamine reuptake, during exposure in early postnatal life [226].

Another molecule to consider in this context is the enzyme monoamine oxidase (MAO), which is responsible for dopamine oxidative metabolism and is localized on the outer mitochondrial membrane [216]. Reversible MAO inhibitors, such as 1-Methyl-1,2,3,4-tetrahydroisoquinoline (1MeTIQ), were evaluated in preclinical studies and compared to olanzapine after pre-treatment with ketamine [227]. In particular, 1MeTIQ, similar to olanzapine, showed to reverse ketamine-induced anxiogenic effects in the elevated plus maze (EPM) test, along with significant inhibition of the dopamine MAO-dependent oxidation pathway [227]. Moreover, 1MeTIQ could increase the concentration of biogenic amines by blocking their catabolism through the MAO-dependent oxidation pathway while preserving their methylation through the action of catechol-O-methyltransferase (COMT) [227]. In this regard, 1MeTIQ was seen to increase the concentration of 3-methoxytyramine (3-MT), an extraneuronal metabolite of dopamine produced by COMT activity [227]. 3-MT was shown to regulate the activity of the catecholaminergic system and antagonize amphetamine-induced behavioral and neurochemical effects [228].

## 7. Mitochondrial Dysfunction, Synaptic Modulation, and Antipsychotic Treatment: Implication for Schizophrenia

Focusing on changes induced, directly or indirectly, by oxidant agents on synapse’s structure and function, a multilevel “locus” of action could be conceived from presynaptic to postsynaptic sites where all antipsychotics exert their action even with different degrees, mainly related to the receptor profile and dose of the drug [229]. Starting with the presynaptic button and the molecular machinery of the presynaptic site, the most recent conceptualization of mitochondria function is the time- and space-dependent regulation of presynaptic energy, or “synaptoenergetics” [230]. Despite the high motility of mitochondria at axon/dendritic branch terminals, the pool of presynaptic mitochondria could play the role of “local power plants” to supply ATP-dependent energy expenditure requirements in a specific presynaptic environment and provide calcium concentrations necessary to enable communication between neurons [231].

Presynaptic dopaminergic molecules influenced by oxidative stress and mitochondrial function include DAT, which is responsible for the clearance of dopamine into the synaptic cleft of the striatum [232]. Specifically, increases in oxidative stress markers, such as 5-S-cysteinyl-dopamine and 5-S-cysteinyl-DOPAC, were found in transgenic mice overexpressing DAT, resulting in neuronal cell death [233]. At the presynaptic level, DAT-mediated reuptake of dopamine could trigger oxidative toxicity and neuronal damage by creating an oxidant environment with elevated levels of ROS, quinones, and toxic intermediates through several mechanisms, including increased mitochondrial MAO activity, autoxidation, and metabolism of dopamine [233,234]. Dopamine oxidation products can damage lipids, DNA, and proteins, including DAT, which is rich in cysteine residues, inducing an “oxidative loop” [235]. Although all available antipsychotics have a very low affinity or no affinity for DAT, there is a growing interest in the role of this molecule in the overall action of antipsychotics, with specific emphasis on TRS [236]. It has been postulated that in the presence of no response to antipsychotics, the blockade or occupancy of DAT by pharmacological agents with relatively high affinity for this transporter may reset the dopaminergic tone by increasing dopaminergic concentration in the synaptic cleft and possibly the response of the dopaminergic system to antipsychotics’ action [237]. In addition, the observance of improvements in rat psychotic-like manifestations after the augmentation of haloperidol with the DAT blocker GBR12909 could rely not only on a “reset” of the dopaminergic tone but also on reduced levels of presynaptic dopamine, ensuing in a low oxidative state [238]. Alterations in DAT function, induced by both pharmacological compounds and oxidative stress, may result in increased dopamine concentrations in the subcortical regions of the brain, especially in the striatum, with a potential double-edged sword effect: (1) contributing to a hyperdopaminergic status, consistent with the dopamine hypothesis of schizophrenia [239]; (2) restoring the dopaminergic tone with a putative beneficial impact on antipsychotics’ efficacy [237].

At the presynaptic glutamatergic site, the excitatory amino acid transporter 3 (EAAT-3), a regulator of synaptic glutamate concentrations, and oxidative stress have also been associated and implicated in antipsychotics’ responsiveness. In particular, Afshari et al. suggested that partial loss of the *Slc1a1* gene, encoding for EAAT-3, in mice, causes haploinsufficiency associated with oxidative stress-induced behavioral, histological, and biochemical changes, promoting the expression of schizophrenia-like features [240]. Pharmacogenomic studies identified an association between the SNP rs16921385 in the *Slc1a1* gene and the response to risperidone treatment, linking oxidative balance and antipsychotic outcomes via synaptic function [241].

At the postsynaptic level, the PSD is a major structure whose components can be affected by oxidative stress with significant pathophysiological consequences and possible clinical relevance [242]. The PSD is considered a multimodal molecular hub where different signaling inputs may converge and are dispatched at intracellular levels [243]. There is substantial evidence that treatment with antipsychotics in a preclinical setting may affect the gene expression of PSD proteins, potentially impacting the architecture and function of the synapse [1]. Here, we will consider some of the most documented oxidative-stress effects on major PSD proteins.

Disc1 is a scaffold protein involved in the PSD composition, whose genetic variants have been variously related to the pathophysiology of schizophrenia [1]. Disc1 could represent a molecular target relevant to schizophrenia treatment, as suggested by the evidence of a significant increase in its expression levels in the frontal cortex of mice administered with olanzapine and risperidone [244]. The interaction between Disc1 and Glycogen synthase kinase-3 beta (GSK3β) has been identified as a factor possibly involved in cell survivance and viability [245]. During oxidative stress, Translin-associated protein X (TRAX), a signaling protein involved in synaptic plasticity and DNA repair, binds to the Disc1/GSK3β complex [245]. Activation of protein kinase A (PKA) and subsequent phosphorylation of GSK3β lead to the dissociation of TRAX, which can move to the nucleus and facilitate DNA repair [245]. Further, GSK3β is a kinase interacting with Akt, whose functional interaction with D2R and modulation of D2R signaling is relatively well characterized and believed to be relevant for antipsychotics’ mechanism of action [246]. Hyperactivity of D2R may trigger D2R-DISC1 complex formation, which is associated with decreased pAkt-pGSK3β signaling and neurite growth reduction [247]. Aripiprazole and haloperidol effectively prevented neurites’ impairments, but only the D2R partial agonist significantly counteracted the reduced phosphorylation in the Akt/GSK3β pathway [247].

Homer1 is one of the three isoforms (e.g., Homer1, Homer2, and Homer3) among the most abundant proteins at PSD, implicated in shaping the architecture of the dendritic spine, involved in the pathophysiology of behavioral disorders [248] as well as in the effects of antipsychotics upon synaptic gene expression [6,249,250]. Oxidant stress may affect Homer proteins [251]. In this context, the structural and functional modifications of Homer proteins in the NMDAR/AMPAR-dependent signaling machinery at the PSD may be a proxy of the alteration induced by oxidative stress on the dendritic spine [251]. Along with the antipsychotics’ impact on Homer1 demonstrated in multiple animal settings [134,249,250], it would be interesting to explore whether the changes in Homer1 expression induced by both canonical and non-canonical antipsychotic mechanisms of action may mirror changes in oxidative stress biomarkers.

Calcium/calmodulin-dependent protein kinase II (CaMKII) is a multimodal serine/threonine protein kinase found throughout the brain [252] and in multiple subcellular compartments of the neuron, and is one of the most abundant proteins at PSD [253,254]. CaMKII has been associated with schizophrenia both in clinical [255,256] and preclinical settings [257]. Mice with a heterozygous null mutation for CaMKII (α-CaMKII+/−) may show altered executive functioning reminiscent of schizophrenia patients [258]. Furthermore, oxidative stress modulates NMDAR activity on GluN1-GluN2A receptors [259,260], inhibiting CaMKII activity [261]. Considering the role of CaMKII in the synaptic machinery and its localization at the PSD level, the association between antipsychotics, oxidative stress, and CaMKII could be relevant for therapeutic purposes. Indeed, CaMKII is responsive to different psychotropic drug administrations [262], including antipsychotics [263]. Studies in CaMKII KO heterozygous rodents with neonatal ventral hippocampal lesions reminiscent of schizophrenia models show a decrease in CaMKII activity, suggesting that CaMKII is a therapeutic target in schizophrenia. Furthermore, preclinical studies enhancing CaMKII activity have shown a potential treatment for social and cognitive disorders in schizophrenia, showing resistance to antipsychotics [257]. It has been speculated that antipsychotic resistance might be associated with reduced levels of α-CaMKII protein in the striatum [264], also resulting as an effect of exposure to oxidative stress. In line with the role of CamKII in the NMDA-hypofunction, it has been reported that CaMKII is involved in the facilitating effect of clozapine on NMDA-induced inward currents and electrically evoked excitatory postsynaptic currents (EPSCs) in medial prefrontal cortex pyramidal cells [265], probably with the implication of its antioxidant proprieties.

The observation that, at the glutamatergic PSD, Shank proteins may contribute to assembling the NMDAR/PSD-95/nitric oxide synthase-1 (NOS1) complex with the guanylate kinase-associated protein (GKAP) adaptor protein is relevant in terms of antipsychotics’ impact on oxidative pathways [266]. NOS1 binds to PSD-95 through PDZ-PDZ domains, allowing a functional interaction between NMDAR and NOS1, which can be localized spatially close to subcellular microdomains rich in Ca^2+^ influx, which is necessary for enzymatic activation [267]. NO production contributes to oxidative stress pathways by reacting with superoxide anions to produce peroxynitrite (ONOO−) ions, which can induce lipid peroxidation and DNA damage leading to cell death [268]. Of interest, antipsychotic treatment has been demonstrated to be effective in modulating Shank and PSD-95 activity, accounting for potential beneficial effects [1]. In addition, the NMDAR co-agonist D-Serine, which has been proposed as an augmentation strategy in schizophrenia patients [208], has been found to reduce the expression of nitric oxide synthase 1 adaptor protein (NOS1AP) and attenuate the decrease in dendrite branching exerted by NOS1AP overexpression in vitro [269].

In summary, the overall scenario that emerges at the presynaptic and postsynaptic levels is that of multiple molecular “loci” where oxidative stress mechanisms may “hit” with potentially relevant consequences for the pathophysiology of behavioral disorders. Antipsychotics could intercept some of these effects, but caution is needed at present in interpreting the effect of this class of compounds in the reciprocal interaction between PSD proteins and oxidative stress.

## 8. Antipsychotic Treatment Effects on Mitochondrial Function and Intracellular Signaling

Several pieces of evidence have explored the impact of antipsychotic medication on proteins involved in mitochondrial functions and signaling pathways relevant to cell viability and survivance.

Effects of first- and second-generation antipsychotics were evaluated by an in vitro analysis of mitochondrial proteins in the lymphocytes of psychotic patients [270]. The hierarchical clustering dendrogram showed a separate cluster for first- and second-generation compounds, suggesting the possibility of exploiting mitochondrial function parameters as a tool for tailored drug treatment in patients with schizophrenia or bipolar disorder [270]. Olanzapine and risperidone were effective in decreasing mitochondrial maximal respiration [270]. Olanzapine decreased levels of caspase 3 and MFN1, whereas risperidone significantly decreased OPA1 protein levels [270]. Risperidone and haloperidol increased the expression of proteins involved in cell viability by raising the levels of both pro- and anti-apoptotic proteins BAX and Bcl-2, respectively [270]. Clozapine increased the levels of Bcl-2 and reduced the levels of Drp1, another protein involved in mitochondrial fission [270]. Haloperidol significantly decreased the levels of the MCI subunits NDUVF1, NDUVF2, NDUVS1, caspase 3, and Drp1 [270].

The different effects of typical or atypical antipsychotics on mitochondrial gene expression were reported by Choi and coauthors [32]. They studied postmortem liver tissue of schizophrenia patients and found an upregulation of *SOD2, 3-phosphoinositide-dependent protein kinase (PDK1)*, and *NSF Attachment Protein Gamma (NAPG)* and downregulation of 11 mitochondria-related genes, including *D-beta-hydroxybutyrate dehydrogenase (BDH1), acyl-coenzyme A dehydrogenase (ACAMD), Solute carrier family 25 member 47 (SLC25A47)*, and *N-Acetylglutamate synthase (NAGS)* in patients treated with typical antipsychotic compared to atypical antipsychotic treatment [32].

Rosenfeld et al. showed that cellular respiration in cells of the lymphoblastoid line is significantly reduced in patients with schizophrenia than controls. In addition, these patients exhibited greater dopamine-induced inhibition [179]. In this study, it was shown that haloperidol suppresses cellular respiration by interacting with complex I [179]. Dopamine interacted with MCI but at a different site than the antipsychotics [179]. However, both dopamine and antipsychotic drugs could inhibit MCI, resulting in possible lower O_2_ consumption in nerve terminals and lower adenosine triphosphate (ATP) production [136,271,272]. Data from the Rosenfeld’s experiments confirmed the previous observations that showed an inhibition in MCI exerted by haloperidol, chlorpromazine, and thiothixene in rat brain mitochondria [179]. The same effect was observed at very high doses of clozapine. Intriguingly, the platelet MCI activity in patients treated with haloperidol and chlorpromazine was reduced compared to patients treated with clozapine, probably due to the efficacy dosage used [179]. This activity alteration was also reported in patients affected by Parkinson’s disease, and could be associated with the extrapyramidal side effects, such as also tardive dyskinesia, of these drugs [273,274]. According to these findings, another preclinical study on slices of mouse brain demonstrated the inhibition of MCI by haloperidol, which proved to be the most potent inhibitor of MCI, probably due to a depletion in glutathione levels, followed by chlorpromazine, fluphenazine, and risperidone but not clozapine [275]. Furthermore, a preclinical study in isolated rat liver mitochondria reported a reduction in MCI activity after chlorpromazine, haloperidol, risperidone, and quetiapine treatment, and the effect of the latter two was 2–4 times less than typical neuroleptics while clozapine and olanzapine had only slight effects [276]. These data were confirmed in a clinical study conducted in peripheral blood mononuclear cells of patients affected by schizophrenia, which also reported an increase in lipid peroxidation after haloperidol treatment compared to risperidone or clozapine [277]. In addition, a preclinical study showed that a decrease in MCI activity in mice brains was induced by haloperidol depending on the doses and timing of drug administration with various responses in different brain regions that could explain the different side effects at multiple D2R occupancy levels [278]. Regarding the effects of antipsychotics in different brain regions, flupenthixol reduced MCI activity in the rat striatum, frontal cortex, hippocampus, and cerebellum after chronic administration [279]; these data were also confirmed in humans affected by schizophrenia [280]. Haloperidol was also responsible for a reduction in oxygen consumption and MCI activity in the brain but not in muscle mitochondria that were prevented or partially reverted by SKF38393 (D1R agonist) and quinpirole (D2R agonist) coadministration [281]. In contrast, the reduction in protein synthesis—e.g., NADH-ubiquinone oxidoreductase chain (ND)1, ND4, ND5, mitochondrial cytochrome oxidase subunit (CO)I, COII, and cytochrome B (Cyb)—found in the haloperidol group was not reverted by dopamine agents [281], leading to the conclusion that dopamine pathways did not mediate this effect [281].

A decrease in the levels of subunits of the mitochondria electron transport chain, including complex I (NDUFV1), complex II (Sdha), complex III (Uqcr10), complex IV (Cox15), and complex V (Atp5c1), was reported in the peripheral blood cells of schizophrenia patients compared to healthy controls after administration of higher risk metabolic syndrome-induced clozapine and olanzapine [24]. Additionally, clozapine and olanzapine reduced transcripts and proteins involved in the energy metabolism, ATP levels, and mitochondrial oxygen consumption evaluated in lymphoblastoid cell lines from both patients and controls [24]. The treatment with quetiapine and risperidone, which have a medium risk of inducing metabolic syndrome, was responsible only for a decrease in NDUFV1, Sdha, and Atp5c1 levels [24]. Finally, all three groups, including patients treated with antipsychotics considered at low risk for inducing metabolic syndrome (aripiprazole and ziprasidone), subjects undergoing treatment with medium-risk second-generation antipsychotics (risperidone and quetiapine), and patients administered with high-risk drugs (clozapine and olanzapine), showed reduced levels of genes coding for proteins involved in mitochondrial fission processes compared to controls [24].

Quetiapine is responsible for mitochondrial swelling and cell membrane destruction, inducing necroptosis by receptor-interacting serine/threonine-protein kinase (RIP) activation and phosphorylation of mixed lineage kinase domain-like protein (MLKL) [282,283]. Other findings have shown that quetiapine also has an antioxidant, anti-inflammatory, and neuroprotective role via the reduction of lipid peroxidation [284,285].

Clozapine-related cardiotoxic effects seem to engage cardiac mitochondria that are involved in the conversion into clozapine reactive metabolites leading to a reduction of oxygen consumption rate [286]. Similarly, the diabetogenic effect exerted by clozapine, studied in β-cells culture, could be related to increases in mitochondrial membrane fluidity and polyunsaturated fatty acid content, resulting in apoptosis and suppression of cell proliferation [287,288]. On the other hand, clozapine exhibited neuroprotective effects through an uncanonical mechanism of action probably mediated by an increase in BDNF levels via Akt-GSK3 cascade activation that reverts oxidative stress damage [289]. Clozapine was also seen to enhance oxidative phosphorylation and galactocerebroside expression, improving neural energy supply [25].

A recent in vitro study has addressed the effects of antipsychotics of more recent introduction in therapy, such as brexpiprazole, cariprazine, loxapine, and lurasidone, and showed impairments in pig brain mitochondria, concluding that these antipsychotics reduced ATP production and the function of electron transport chain enzymes, except for complex IV, whose activity was found increased after brexpiprazole and loxapine addition [290].

The effects of antipsychotics on mitochondrial function are sufficiently proven, but little is still known about the mechanisms of action of antipsychotics on mitochondria responsible for their effects. Chan and colleagues have hypothesized three different possibilities: (i) influence on ionic membrane permeability; (ii) binding of antipsychotics at G protein-coupled receptors (GPCRs) alters protein kinase A (PKA) or PKC levels modifying their binding at mitochondrial membrane; (iii) direct binding of antipsychotics to the mitochondrial membrane [291]. To better understand the antipsychotics’ effects, two proteomics studies were conducted by the same group to analyze their effects on intracellular pathways. A preclinical study investigated the different effects of the chronic administration of chlorpromazine, clozapine, or quetiapine on the modulation of mitochondrial proteins, mainly from the oxidative phosphorylation pathways in the rat cortex and hippocampus [292]. Three antipsychotic treatments, such as chlorpromazine, clozapine, and quetiapine, modulated the synaptic proteome in a rat cerebral cortex by regulating proteins related to the energy production or the glycolytic pathways, as well as to the G-protein-coupled signal transduction system [293].

**Figure 3:**
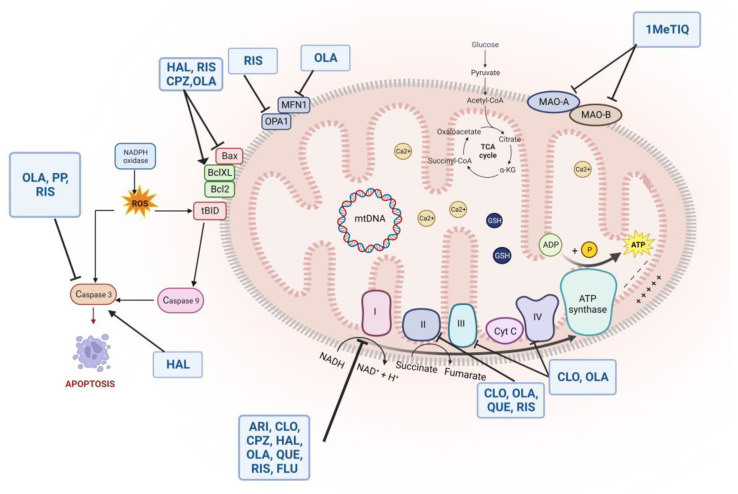
Antipsychotics can interact with several proteins involved in the regulation of mitochondrial functions, including energy expenditure, free radicals’ production, and cell survivance. For a comprehensive acknowledgment of antipsychotics’ sites of action at the mitochondrial level, please see the corresponding text. 1MeTIQ = 1-Methyl-1,2,3,4-tetrahydroisoquinoline; ARI = aripiprazole; CLO = clozapine; CPZ = chlorpromazine; FLU = fluphenazine; GSH = glutathione; HAL = haloperidol; MAO = monoamine oxidase; MFN1 = mitofusin 1; OLA = olanzapine; OPA1 = optic atrophy 1; QUE = quetiapine; PP = paliperidone; RIS = risperidone. Created with BioRender.com; last accessed on 23 February 2023.

These results may suggest that antipsychotics could modify mitochondrial function impairing their oxygen consumption and energy production, but, on the other hand, are also able to rebalance altered oxidative stress related to the neurobiological basis of psychiatric diseases via different intracellular pathways, resulting in their neuroprotective effects [291,294]. Putative mitochondrial sites of interest for antipsychotics’ action have been outlined in Figure 3.

## 9. Antioxidants: A Possible Add-on Strategy in the Treatment of Schizophrenia?

Several RCTs have been conducted to assess the efficacy of antioxidant strategies, including Ginkgo biloba, NAC, allopurinol, vitamin C, and vitamin E, as add-on treatments to standard antipsychotic medication [295].

Allopurinol was studied in combination with haloperidol and showed a significant superiority over the antipsychotic alone in the treatment of positive symptoms and the PANSS total score [296]. In a crossover clinical trial, allopurinol showed safety, tolerability, and significant improvements in the PANSS total, positive, negative, and general scores compared to the placebo [297]. In an 8-week RCT, no differences were found in the PANSS or cognitive measures between groups treated with allopurinol or placebo in augmentation to antipsychotics [298]. In another trial, patients treated with allopurinol rated themselves as more improved than the placebo group [299].

The efficacy of Ginkgo biloba was evaluated in RCTs conducted on patients diagnosed with schizophrenia and showed to significantly decrease SANS and Scale for the Assessment of Positive Symptoms (SAPS) scores when added to haloperidol and administered at a dose of 360 mg/day [300]. In chronic schizophrenia, the administration of Ginkgo biloba was effective in ameliorating brief psychiatric rating scale (BPRS) and SANS scores compared to the placebo [301,302].

Vitamin E supplementation was evaluated as an augmentation strategy in schizophrenia patients under treatment with haloperidol, and even if no difference was detected in the PANSS total or subscale scores, the concentration of oxidized glutathione was decreased while the activity of catalase and superoxide dismutase was increased [303].

After vitamin C supplementation in schizophrenia patients treated with second-generation antipsychotics, blood levels of malondialdehyde and ascorbic acid were significantly increased compared to the placebo, together with an improvement in the BPRS score [304]. In one study, the simultaneous administration of vitamin C and E was found to worsen psychotic symptoms, especially persecutory delusions, in patients with low blood levels of polyunsaturated fatty acids (PUFA). In contrast, they had no detrimental effect on psychosis in patients with high PUFA levels [305].

Among antioxidants, NAC has been the most investigated compound in psychotic disorders by RCTs. In patients with chronic schizophrenia, the administration of 1 g twice daily of NAC as an add-on treatment was significantly associated with a reduction in the PANSS total, negative, and general scores as well as an improvement in akathisia [306]. After 8-week treatment, the augmentation of risperidone with NAC significantly decreased PANSS total and negative subscale scores compared to placebo without any difference in the frequency of adverse effects [307]. NAC-treated patients also exhibited a significant improvement in cognitive functions. Specifically, in individuals with chronic schizophrenia, NAC significantly ameliorated PANSS negative and positive scores, along with improvement in attention, short-term and working memory, executive functioning, and speed of processing [308]. Improvement in working memory after NAC add-on treatment was replicated in another study conducted on psychotic patients, including individuals affected by schizophrenia or bipolar disorder [309]. The effect on illness duration was evaluated in light of NAC responsiveness, showing that patients with chronic schizophrenia might benefit more than others from additional treatment with NAC in terms of functioning and positive symptoms [310]. In psychotic patients, NAC administration was also associated with improvement in mismatch negativity, measured by auditory evoked potentials and whose amplitude is commonly reduced in schizophrenia, even though no difference in the PANSS total or subscale scores was detected in comparison to placebo [311]. Moreover, NAC effectively modulated functional connectivity in patients diagnosed with schizophrenia by increasing the multivariate phase synchronization over the left parietal-temporal, the right temporal, and the bilateral prefrontal regions, as assessed by EEG application [312]. Six-week add-on treatment with NAC in early psychosis patients effectively ameliorates processing speed and positive symptoms, along with an increase in white matter integrity in the fornix measured by generalized fractional anisotropy [313]. Further, NAC supplementation in early psychosis patients was significantly associated with an elevation in functional connectivity within the cingulate cortex, with an increased node betweenness centrality value in the isthmus of the cingulate cortex [314]. The cingulate cortex, a key component of the salience network, has been found altered in psychotic patients [315], and betweenness centrality measures of nodes comprised in this region were significantly modulated by second-generation antipsychotics in preclinical models of schizophrenia [249]. Treatment with NAC was evaluated over a 52-week period in early-phase psychotic disorders. It showed to reduce the PANSS total, negative, and disorganized thought scores without any change in PANSS positive and cognitive scores as well as no impact on brain morphology, as measured by magnetic resonance imaging (MRI) scans [316]. The effect on disorganized thought is relevant in prognostic terms since disorganization domain scores may be predictors of treatment-resistant schizophrenia [317]. In a placebo-controlled RCT for TRS patients with persistent psychotic symptoms treated with clozapine, NAC as adjuvant therapy was not shown to significantly improve cognitive symptoms or quality of life in TRS patients [318]. Furthermore, a recent meta-analysis inferred that the recommendation in routine clinical practice for NAC treatment in schizophrenia might be premature despite its antioxidant potential. However, the results suggest evaluating new avenues of therapeutic approach in which NAC with a dosage ≥ 2000 mg/d for at least 6 months could potentially improve functional outcomes in schizophrenia [319]. To highlight differences in the efficacy and tolerability of compounds with a main antioxidant effect, perhaps due to the different antipsychotic drugs to which they were added, RCTs specifically reporting the type of therapy at baseline were collected in Table 2.

Multiple studies have evaluated the ability of antioxidant agents to ameliorate the side effects of antipsychotics, especially with regard to tardive dyskinesia, a complication resulting from the long-term use of D2R blockers. When provided in individuals affected by both schizophrenia and tardive dyskinesia at a dose of 240 mg/day, Ginkgo biloba was effective in reducing the Abnormal Involuntary Movement Scale (AIMS) scores but not psychotic or cognitive symptoms [320]. Although in one study, the administration of vitamin E was not effective in the treatment of dyskinetic movements [321], in another sample of patients with tardive dyskinesia and schizophrenia, vitamin E was more effective than placebo at reducing the AIMS score along with an increase in blood SOD levels, but not to ameliorate psychotic manifestations [322]. The other two studies replicated the reduction in the AIMS score induced by vitamin E compared to the placebo [323,324]. To sum up, it should be recognized that, at present, the evidence regarding the efficacy of vitamin E in tardive dyskinesia is still to be fully clarified.

**Table 2:** RCTs of antioxidant agents as add-on treatment in patients with psychotic disorders.

Type of Antioxidant	Antipsychotics	Main Outcomes	References
Allopurinol	Haloperidol (46 patients with SCZ)	There was a significant superiority over the antipsychotic alone in the treatment of positive symptoms and PANSS total score.	Akhondzadeh et al., 2005 [296]
	Olanzapine (13 patients) Risperidone (13 patients) Clozapine (18 patients) Quetiapine (5 patients) Aripiprazole (4 patients) Haloperidol (7 patients) Patients with SCZ enrolled: 59	Patients treated with allopurinol rated themselves as more improved than the placebo group.	Dickerson et al., 2009 [299]
Ginkgo biloba	Haloperidol (109 patients with SCZ)	EGb significantly decreased SANS and SAPS scores when added to haloperidol.	Zhang et al., 2001 [300]
	Clozapine (42 patients with TRS)	EGb, in addition to clozapine, improves negative symptoms in patients with TRS.	Doruk et al., 2008 [302]
Vitamin E and EPUFAs	Haloperidol decanoate depot injection (52 patients with SCZ)	Vitamin E decreased GSSG concentration and motor delay on the overall scale of PANSS, while EPUFAs increased GSH.	Bošković et al., 2016 [303]
Vitamin E	Fluphenazine decanoate (40 patients with SCZ and TD)	The reduction in AIMS score induced by vitamin E in comparison to placebo.	Adler et al., 1998 [324]
Vitamin C	Olanzapine (10 mg/day) or quetiapine (200 mg/day) or ziprasidone (40 mg/day). Patients with SCZ enrolled: 40	After vitamin C supplementation in patients treated with second-generation antipsychotics, blood levels of malondialdehyde and ascorbic acid were increased compared to the placebo, with an improvement in the BPRS score.	Dakhale et al., 2005 [304]
NAC	Clozapine (45% of participants) and olanzapine (20% of participants), other atypical antipsychotics (risperidone, quetiapine, and aripiprazole), and typical depot antipsychotics. Patients enrolled: 140	The administration of 1 g twice daily of NAC as an add-on treatment was associated with a reduction in PANSS total, negative, and general scores as well as an improvement in akathisia.	Berk et al., 2008 [306]
	Risperidone (up to 6 mg/d) for 8 weeks	NAC decreased the PANSS total and negative scores compared to the placebo without any difference in the frequency of adverse effects.	Farokhnia et al., 2013 [307]
	Chlorpromazine equivalent to 300–1000 mg (except clozapine). Patients with SCZ enrolled: 84	NAC-treated patients exhibited an improvement in cognitive functions and ameliorated PANSS negative and positive scores, along with improvement in attention, short-term and working memory, executive functioning, and speed of processing.	Sepehrmanesh et al., 2018 [308]
	Clozapine, olanzapine, and other antipsychotics. Patients with SCZ enrolled: 121	Patients with SCZ might benefit more than others from additional treatment with NAC in terms of functioning and positive symptoms.	Rapado-Castro et al., 2017 [309]
	Risperidone, quetiapine, clozapine, olanzapine. Patients with SCZ enrolled: 11	NAC administration was associated with improvement in mismatch negativity, measured by auditory evoked potentials, and there was no difference in the PANSS total or subscale scores in comparison to the placebo.	Lavoie et al., 2008 [311]
	Quetiapine (8 patients) Clozapine (1 patient) Aripiprazole (3 patients) Amisulpride (2 patients) Risperidone (3 patients) Olanzapine (2 patients). Patients with SCZ enrolled: 20	Six-week add-on treatment with NAC in early psychosis patients was effective in ameliorating processing speed and positive symptoms, along with an increase in white matter integrity in the fornix measured by generalized fractional anisotropy.	Klauser et al., 2018 [313]
	Clozapine Patients with enduring psychotic symptoms enrolled: 84	NAC, administered at 2 g/day, was not effective in improving negative symptoms, cognition, and quality of life at 8, 24, and 52 weeks in TRS patients taking clozapine.	Neill et al., 2022 [318]

AIMS = Abnormal Involuntary Movements Scale; BPRS= brief psychiatric rating scale; EGb = ginkgo biloba; EPUFAs = essential polyunsaturated fatty acids; GSH = glutathione; GSSG = glutathione disulfide; NAC = N-acetylcysteine; PANSS = Positive and Negative Syndrome Scale; SANS = Scale for the Assessment of Negative Symptoms; SAPS = Scale for the Assessment of Positive Symptoms; SCZ = schizophrenia; TD = tardive dyskinesia; TRS = treatment-resistant schizophrenia.

## 10. Discussion

Several lines of evidence suggest a possible involvement of oxidative stress in schizophrenia pathogenesis in terms of both abnormal brain development [77] and peri/postnatal neurotoxic damage [26]. Antipsychotics are the mainstay of schizophrenia pharmacological treatment [325], and their mechanisms of action have been demonstrated to putatively intercept a few of the factors believed relevant for the onset or progression of the disease, including the oxidative balance [326]. Alterations in oxidative stress impair the regulation of the release of major neurotransmitters implicated in the disorder, above all dopamine [327] and glutamate [58], and their intracellular signaling both at cortical and subcortical levels [107,108,109], as well as the modification of the synapse structure and function [58]. Although alterations in oxidative pathways are observed in psychotic patients, it remains elusive to conclude whether this is an epiphenomenon secondary to alterations in dopaminergic neurotransmission or a primary aspect directly related to the development of clinical symptoms. Observing psychotic-like behaviors in animal models of neuroinflammation and oxidative stress-related toxicity suggests that these factors could be primary aspects of the pathophysiology of schizophrenia and other psychotic disorders [328].

Despite the growing evidence pointing to the role of antipsychotics in counterbalancing oxidative stress and mitochondria dysfunction, several aspects should be considered in a critical appraisal of the issue.

First, evidence point to the antioxidant effects of antipsychotics, but is this action a relevant one for the overall antipsychotic effect? There is little evidence that the involvement of antipsychotics in aberrant oxidative process and/or mitochondrial dysfunction have a direct primary antipsychotic effect. On the other side, the attenuation or reversal of a few markers of oxidative stress and mitochondrial dysfunction could have at least in part a protective role in preserving the structure and function of the synapse [77].

Second, whereas the evidence of a possible increased inflammatory index in schizophrenia patients and changes after antipsychotics have been reported [329], there is a lack of information on the in vivo effects of antipsychotics on the inflammasome associated specifically with oxidative stress and free radicals’ production [330,331]. Therefore, more studies investigating peripheral biomarkers of oxidative stress before and after antipsychotic treatment should be implemented. Enforcing this strategy could be helpful to understand the weight of antipsychotic effects on oxidative stress markers as well as the impact of the redox balance on treatment response. This perspective is specifically relevant for disentangling, even with the limits of peripheral markers, the putative contribution or association of oxidative stress in the response or resistance to antipsychotic treatment [332,333].

Third, antipsychotics, especially first-generation compounds, have been associated with both reducing (or counterbalancing) and increasing oxidative stress markers [187]. This controversial aspect calls for more stringent research and appraisal of this potential dichotomic issue. At present, the most conservative approach is probably to consider the impact of antipsychotics on oxidative mechanisms not as a “class effect” but as an effect dependent on the receptor profile, dose of the antipsychotic, experimental model, brain region or body tissue, and specific components of the oxidative stress dependent-signaling [179,187,202,213]. Among the most promising next-generation antipsychotics, xanomeline, a preferential muscarinic M1R and M4R agonist has been explicitly texted for protection against oxidative stress [334]. It should be considered that the actual antipsychotic for schizophrenia therapy is a combination of xanomeline and trospium, with the latter to prevent peripheral adverse events of muscarinic agonism [334]. Exposure of primary rat neuronal cells to xanomeline alone effectively protected the cells from oxygen-glucose deprivation (OGD)-induced injury and cell apoptosis, as well as inhibiting ROS build-up induced by OGD and preventing p-Akt reduction [215].

Fourth, there is a growing interest in expanding the action of antipsychotics with the addition/combination of antioxidants. This is a strategy worth pursuing, but caution is needed in drawing conclusions at this time. Indeed, while the translational background is solid and the strategy overall viable, the pilot and preliminary studies conducted to date have shown mixed results [295]. Vitamin E and C were almost ineffective on psychotic symptoms, whereas they ameliorated dyskinetic movements in patients treated with antipsychotics [322,323,324]. On the other hand, it would be important to conceptualize potential biomarkers of putative theranostic value that could give guidance on the effect of the antioxidant agent add-on. In this line, few but promising original results and proofs of concept have been reported for NAC, an antioxidant and glutathione precursor, which has been to improve cognitive and psychotic manifestations, along with modifications in functional connectivity and white matter integrity detected in the cingulate cortex and fornix, respectively [306,313,314].

Fifth, by means of induced pluripotent stem cells from patients with TRS, it has been shown that mitochondrial alterations related to neuregulin-1 deficiency were significantly associated with olanzapine treatment outcomes and that miR143-3p levels correlated with the severity of olanzapine resistance [100]. Mitochondrial proteomic studies as well as investigation of specific patterns of mitochondrial functions and components, showed a unique and distinct effect for each antipsychotic [270,292,293]. In addition, schizophrenia patients with different PUFA blood levels showed different responses to antioxidant compounds added to antipsychotic medication [305].

These results suggest an opportunity to further explore antioxidant mechanisms and the reciprocal interaction with antipsychotic action.

### Limitations

Several limitations must be taken into account. Most RCTs have been conducted by adding antioxidant agents to different antipsychotic treatments (Table 2). The patients included in the various studies also had different diagnoses, including chronic schizophrenia, early psychosis, and bipolar disorder [306,309,310,313]. Thus, it is difficult to compare the effects of different compounds on psychotic symptoms without proper stratification of baseline treatment and diagnosis.

Only a few studies have explored the role of antioxidant agents in modifying functional connectivity parameters and the inflammatory status of patients affected by schizophrenia, along with the improvement of psychotic symptoms [314].

The evidence obtained was insufficient to clearly establish an effect of disease course, antipsychotic dosage, and other comorbidities on the antioxidant properties of the compounds studied. In particular, the doses of antipsychotics were within a wide range even in the same study [31].

In preclinical models, the antioxidant effects of various antipsychotics were evaluated considering the distinction between typical and atypical drugs but without adequately separating the data obtained from the specific pharmacodynamic profile of each compound with regard to the receptor and intracellular pathways involved. Further, the antioxidant effect of antipsychotics has not been compared with that of other CNS drugs, such as antiepileptics, antidepressants, sedatives, and anti-Parkinson’s drugs.

Lastly, it remains to determine whether the antioxidant effect of antipsychotics by itself constitutes a mechanism of action associated with the clinical benefit of psychotic symptoms or whether it is just an epiphenomenon due to the impact of drugs on neurotransmitter pathways, especially the dopaminergic one.

## 11. Conclusions

In conclusion, multiple lines of evidence suggest a role for oxidative stress and mitochondria dysfunction in the pathogenesis and pathophysiology of schizophrenia. Preclinical findings point to the involvement of unbalanced redox mechanisms in dopaminergic and glutamatergic signaling and in synaptic function, setting the scene for investigating the intersection of antipsychotics’ effect with redox mechanisms.

Albeit with caution, preclinical and clinical data suggest an antioxidant role for antipsychotic drugs that could also contribute to their clinical efficacy through modulation of synaptic plasticity and functional connectivity processes [238,244,314].

The impact of antipsychotics on oxidative mechanisms should not be considered a class effect since differences between typical and atypical compounds were found, with a major antioxidant effect exerted by the latter and controversial evidence referred to the former [29,190,211]. Novel compounds, such as xanomeline, have recently shown promising antipsychotic efficacy along with protection against oxidative stress [215]. The observation of antioxidant properties exhibited by atypical agents with different pharmacodynamic profiles suggests the possibility that mechanisms other than the receptor one are involved in the modulation of oxidative stress by antipsychotics, such as drug metabolism at the neuronal level or action on enzymes involved in the free radical generation [29,203].

Although NAC has shown positive results in reducing psychotic symptoms, more appropriate stratification of patients by baseline therapy and clinical diagnosis will be needed to compare the effects of antioxidant compounds and their ability to modulate neurobiological outcomes [294,297,298,301].

More studies on the precise role of oxidants and mitochondria in the pathophysiology of schizophrenia, as well as of antioxidants alone or in combination with antipsychotics for the treatment of the disorder, are warranted.

## Data Availability

Not applicable.

## Supplementary Materials

The search strings for each database, PRISMA checklist, and study design of all the articles included in the present systematic review can be downloaded at: https://www.mdpi.com/article/10.3390/antiox12040975/s1.
