# Supplementary material for: Schizophrenia Synaptic Pathology and Antipsychotic Treatment in the Framework of Oxidative and Mitochondrial Dysfunction: Translational Highlights for the Clinics and Treatment
Source: Antioxidants (Basel). 2023 Apr 21;12(4):975. doi: 10.3390/antiox12040975 (PMC10135787; doi:10.3390/antiox12040975)
Supplement: Supplementary file 1 [file antioxidants-12-00975-s001.zip › antioxidants-2305599-supplementary.pdf]

Supplementary materials

Search strings:

Pubmed (1433) ((((("schizophrenia"[Title/Abstract]) OR ("schizophrenia spectrum and other psychotic disorders"[MeSH Terms])) OR ("psychosis"[Title/Abstract])) OR ("psychotic disorder"[Title/Abstract])) OR ("antipsychotic"[Title/Abstract])) AND ((((("mitochondria"[Title/Abstract]) OR (mitochondrion[Title/Abstract])) OR ("mitochondria"[MeSH Terms])) OR ("free radical"[Title/Abstract])) OR ("antioxidant"[Title/Abstract]))

Embase (1906) #1 AND #2

#2

mitochondria:ti,ab,kw OR mitochondrion:ti,ab,kw OR 'free radical':ti,ab,kw OR antioxidant:ti,ab,kw

#1

schizophrenia:ti,ab,kw OR psychosis:ti,ab,kw OR antipsychotic:ti,ab,kw OR 'psychotic disorder':ti,ab,kw

Scopus (3027)

((TITLE-ABS-KEY (schizophrenia) OR TITLE-ABS-KEY (psychosis) OR TITLE-ABS-KEY (psychotic AND disorder) OR TITLE-ABS-KEY (antipsychotic))) AND ((TITLE-ABS-KEY (mitochondria) OR TITLE-ABS-KEY (mitochondrion) OR TITLE-ABS-KEY (free AND radical) OR TITLE-ABS-KEY (antioxidant)))

Section and Topic	Item #	Checklist item	Location where item is reported
**TITLE**			
Title	1	Identify the report as a systematic review.	1
**ABSTRACT**			
Abstract	2	See the PRISMA 2020 for Abstracts checklist.	1
**INTRODUCTION**			
Rationale	3	Describe the rationale for the review in the context of existing knowledge.	2
Objectives	4	Provide an explicit statement of the objective(s) or question(s) the review addresses.	2-3
**METHODS**			
Eligibility criteria	5	Specify the inclusion and exclusion criteria for the review and how studies were grouped for the syntheses.	3
Information sources	6	Specify all databases, registers, websites, organisations, reference lists and other sources searched or consulted to identify studies. Specify the date when each source was last searched or consulted.	3
Search strategy	7	Present the full search strategies for all databases, registers and websites, including any filters and limits used.	3
Selection process	8	Specify the methods used to decide whether a study met the inclusion criteria of the review, including how many reviewers screened each record and each report retrieved, whether they worked independently, and if applicable, details of automation tools used in the process.	3
Data collection process	9	Specify the methods used to collect data from reports, including how many reviewers collected data from each report, whether they worked independently, any processes for obtaining or confirming data from study investigators, and if applicable, details of automation tools used in the process.	3
Data items	10a	List and define all outcomes for which data were sought. Specify whether all results that were compatible with each outcome domain in each study were sought (e.g. for all measures, time points, analyses), and if not, the methods used to decide which results to collect.	3
	10b	List and define all other variables for which data were sought (e.g. participant and intervention characteristics, funding sources). Describe any assumptions made about any missing or unclear information.	3
Study risk of bias assessment	11	Specify the methods used to assess risk of bias in the included studies, including details of the tool(s) used, how many reviewers assessed each study and whether they worked independently, and if applicable, details of automation tools used in the process.	Not applicable
Effect measures	12	Specify for each outcome the effect measure(s) (e.g. risk ratio, mean difference) used in the synthesis or presentation of results.	Not applicable
Synthesis methods	13a	Describe the processes used to decide which studies were eligible for each synthesis (e.g. tabulating the study intervention characteristics and comparing against the planned groups for each synthesis (item #5)).	Not applicable
	13b	Describe any methods required to prepare the data for presentation or synthesis, such as handling of missing summary statistics, or data conversions.	Not applicable
	13c	Describe any methods used to tabulate or visually display results of individual studies and syntheses.	Not applicable
	13d	Describe any methods used to synthesize results and provide a rationale for the choice(s). If meta-analysis was performed, describe the model(s), method(s) to identify the presence and extent of statistical heterogeneity, and software package(s) used.	Not applicable
	13e	Describe any methods used to explore possible causes of heterogeneity among study results (e.g. subgroup analysis, meta-regression).	Not

Section and Topic	Item #	Checklist item	Location where item is reported
			applicable
	13f	Describe any sensitivity analyses conducted to assess robustness of the synthesized results.	Not applicable
Reporting bias assessment	14	Describe any methods used to assess risk of bias due to missing results in a synthesis (arising from reporting biases).	Not applicable
Certainty assessment	15	Describe any methods used to assess certainty (or confidence) in the body of evidence for an outcome.	Not applicable
**RESULTS**			
Study selection	16a	Describe the results of the search and selection process, from the number of records identified in the search to the number of studies included in the review, ideally using a flow diagram.	4
	16b	Cite studies that might appear to meet the inclusion criteria, but which were excluded, and explain why they were excluded.	Not applicable
Study characteristics	17	Cite each included study and present its characteristics.	4-28
Risk of bias in studies	18	Present assessments of risk of bias for each included study.	Not applicable
Results of individual studies	19	For all outcomes, present, for each study: (a) summary statistics for each group (where appropriate) and (b) an effect estimate and its precision (e.g. confidence/credible interval), ideally using structured tables or plots.	Not applicable
Results of syntheses	20a	For each synthesis, briefly summarise the characteristics and risk of bias among contributing studies.	Not applicable
	20b	Present results of all statistical syntheses conducted. If meta-analysis was done, present for each the summary estimate and its precision (e.g. confidence/credible interval) and measures of statistical heterogeneity. If comparing groups, describe the direction of the effect.	Not applicable
	20c	Present results of all investigations of possible causes of heterogeneity among study results.	Not applicable
	20d	Present results of all sensitivity analyses conducted to assess the robustness of the synthesized results.	Not applicable
Reporting biases	21	Present assessments of risk of bias due to missing results (arising from reporting biases) for each synthesis assessed.	Not applicable
Certainty of evidence	22	Present assessments of certainty (or confidence) in the body of evidence for each outcome assessed.	Not applicable
**DISCUSSION**			
Discussion	23a	Provide a general interpretation of the results in the context of other evidence.	29-30
	23b	Discuss any limitations of the evidence included in the review.	29-30
	23c	Discuss any limitations of the review processes used.	Not applicable

Section and Topic	Item #	Checklist item	Location where item is reported
	23d	Discuss implications of the results for practice, policy, and future research.	29-30
**OTHER INFORMATION**			
Registration and protocol	24a	Provide registration information for the review, including register name and registration number, or state that the review was not registered.	3
	24b	Indicate where the review protocol can be accessed, or state that a protocol was not prepared.	3
	24c	Describe and explain any amendments to information provided at registration or in the protocol.	3
Support	25	Describe sources of financial or non-financial support for the review, and the role of the funders or sponsors in the review.	30
Competing interests	26	Declare any competing interests of review authors.	30
Availability of data, code and other materials	27	Report which of the following are publicly available and where they can be found: template data collection forms; data extracted from included studies; data used for all analyses; analytic code; any other materials used in the review.	Not applicable

Prisma checklist

From: Page MJ, McKenzie JE, Bossuyt PM, Boutron I, Hoffmann TC, Mulrow CD, et al. The PRISMA 2020 statement: an updated guideline for reporting systematic reviews. BMJ 2021;372:n71. doi: 10.1136/bmj.n71

Supplementary Table S1. Studies included in the systematic review.

Author	Title	Study design
Abdalla, D. S.-//-Bechara, E. J.	The effect of chlorpromazine and Li2CO3 on the superoxide dismutase and glutathione peroxidase activities of rat brain, liver and erythrocytes	preclinical
Abdel-Salam, O. M.-//-El-Sayed El-Shamarka, M.-//-Salem, N. A.-//-El-Mosallamy, A. E.-//-Sleem, A. A.	Amelioration of the haloperidol-induced memory impairment and brain oxidative stress by cinnarizine	preclinical
Abou El-Magd, R. M.-//-Park, H. K.-//-Kawazoe, T.-//-Iwana, S.-//-Ono, K.-//-Chung, S. P.-//-Miyano, M.-//-Yorita, K.-//-Sakai, T.-//-Fukui, K.	The effect of risperidone on D-amino acid oxidase activity as a hypothesis for a novel mechanism of action in the treatment of schizophrenia	preclinical
Adler, L. A.-//-Peselow, E.-//-Rotrosen, J.-//-Duncan, E.-//-Lee, M.-//-Rosenthal, M.-//-Angrist, B.	Vitamin E treatment of tardive dyskinesia	clinical
Adler, L. A.-//-Edson, R.-//-Lavori, P.-//-Peselow, E.-//-Duncan, E.-//-Rosenthal, M.-//-Rotrosen, J.	Long-term treatment effects of vitamin E for tardive dyskinesia	clinical
Afshari, P.-//-Yao, W. D.-//-Middleton, F. A.	Reduced Slc1a1 expression is associated with neuroinflammation and impaired sensorimotor gating and cognitive performance in mice: Implications for schizophrenia	preclinical
Akhondzadeh, S.-//-Safarcherati, A.-//-Amini, H.	Beneficial antipsychotic effects of allopurinol as add-on therapy for schizophrenia: a double blind, randomized and placebo controlled trial	clinical
Altar, C. A.-//-Jurata, L. W.-//-Charles, V.-//-Lemire, A.-//-Liu, P.-//-Bukhman, Y.-//-Young, T. A.-//-Bullard, J.-//-Yokoe, H.-//-Webster, M. J.-//-Knable, M. B.-//-Brockman, J. A.	Deficient hippocampal neuron expression of proteasome, ubiquitin, and mitochondrial genes in multiple schizophrenia cohorts	preclinical
Amiri, S.-//-Jafari-Sabet, M.-//-Keyhanfar, F.-//-Falak, R.-//-Shabani, M.-//-Rezayof, A.	Hippocampal and prefrontal cortical NMDA receptors mediate the interactive effects of olanzapine and lithium in memory retention in rats: the involvement of CAMKII-CREB signaling pathways	preclinical
Andrade, C.	Antipsychotic Augmentation With N-Acetylcysteine for Patients With Schizophrenia	review
Andreazza, A. C.-//-Shao, L.-//-Wang, J. F.-//-Young, L. T.	Mitochondrial complex I activity and oxidative damage to mitochondrial proteins in the prefrontal cortex of patients with bipolar disorder	preclinical
Ansari, Z.-//-Pawar, S.-//-Seetharaman, R.	Neuroinflammation and oxidative stress in schizophrenia: are these opportunities for repurposing?	review
Antkiewicz-Michaluk, L.-//-Ossowska, K.-//-Romańska, I.-//-Michaluk, J.-//-Vetulani, J.	3-Methoxytyramine, an extraneuronal dopamine metabolite plays a physiological role in the brain as	preclinical

	an inhibitory regulator of catecholaminergic activity	
Balijepalli, S.-//-Boyd, M. R.-//-Ravindranath, V.	Inhibition of mitochondrial complex I by haloperidol: the role of thiol oxidation	preclinical
Balijepalli, S.-//-Kenchappa, R. S.-//-Boyd, M. R.-//-Ravindranath, V.	Protein thiol oxidation by haloperidol results in inhibition of mitochondrial complex I in brain regions: comparison with atypical antipsychotics	preclinical
Barrientos, A.-//-Marín, C.-//-Miró, O.-//-Casademont, J.-//-Gómez, M.-//-Nunes, V.-//-Tolosa, E.-//-Urbano-Márquez, A.-//-Cardellach, F.	Biochemical and molecular effects of chronic haloperidol administration on brain and muscle mitochondria of rats	preclinical
Bar-Yosef, T.-//-Hussein, W.-//-Yitzhaki, O.-//-Damri, O.-//-Givon, L.-//-Marom, C.-//-Gurman, V.-//-Levine, J.-//-Bersudsky, Y.-//-Agam, G.-//-Ben-Shachar, D.	Mitochondrial function parameters as a tool for tailored drug treatment of an individual with psychosis: a proof of concept study	preclinical
Ben-Shachar, D.	Mitochondrial dysfunction in schizophrenia: a possible linkage to dopamine	review
Ben-Shachar, D.	Mitochondrial multifaceted dysfunction in schizophrenia; complex I as a possible pathological target	review
Ben-Shachar, D.-//-Karry, R.	Neuroanatomical pattern of mitochondrial complex I pathology varies between schizophrenia, bipolar disorder and major depression	preclinical
Bentsen, H.-//-Osnes, K.-//-Refsum, H.-//-Solberg, D. K.-//-Bøhmer, T.	A randomized placebo-controlled trial of an omega-3 fatty acid and vitamins E+C in schizophrenia	clinical
Bergman, O.-//-Ben-Shachar, D.	Mitochondrial Oxidative Phosphorylation System (OXPHOS) Deficits in Schizophrenia: Possible Interactions with Cellular Processes	review
Berk, M.-//-Copolov, D.-//-Dean, O.-//-Lu, K.-//-Jeavons, S.-//-Schapkaitz, I.-//-Anderson-Hunt, M.-//-Judd, F.-//-Katz, F.-//-Katz, P.-//-Ording-Jespersen, S.-//-Little, J.-//-Conus, P.-//-Cuenod, M.-//-Do, K. Q.-//-Bush, A. I.	N-acetyl cysteine as a glutathione precursor for schizophrenia--a double-blind, randomized, placebo-controlled trial	clinical
Bertholet, A. M.-//-Millet, A. M.-//-Guillermin, O.-//-Daloyau, M.-//-Davezac, N.-//-Miquel, M. C.-//-Belenguer, P.	OPA1 loss of function affects in vitro neuronal maturation	preclinical
Bodhinathan, K.-//-Kumar, A.-//-Foster, T. C.	Intracellular redox state alters NMDA receptor response during aging through Ca2+/calmodulin-dependent protein kinase II	preclinical
Bošković, M.-//-Vovk, T.-//-Koprivšek, J.-//-Plesničar, B. K.-//-Grabnar, I.	Vitamin E and essential polyunsaturated fatty acids supplementation in schizophrenia patients treated with haloperidol	clinical
Boz, Z.-//-Hu, M.-//-Yu, Y.-//-Huang, X. F.	N-acetylcysteine prevents olanzapine-induced oxidative stress in mHypoA-59 hypothalamic neurons	preclinical

Breier, A.-//-Liffick, E.-//-Hummer, T. A.-//-Vohs, J. L.-//-Yang, Z.-//-Mehdiyoun, N. F.-//-Visco, A. C.-//-Metzler, E.-//-Zhang, Y.-//-Francis, M. M.	Effects of 12-month, double-blind N-acetyl cysteine on symptoms, cognition and brain morphology in early phase schizophrenia spectrum disorders	clinical
Brenman, Jay E.-//-Chao, Daniel S.-//-Gee, Stephen H.-//-McGee, Aaron W.-//-Craven, Sarah E.-//-Santillano, Daniel R.-//-Wu, Ziqiang-//-Huang, Fred-//-Xia, Houhui-//-Peters, Matthew F.-//-Froehner, Stanley C.-//-Bredt, David S.	Interaction of Nitric Oxide Synthase with the Postsynaptic Density Protein PSD-95 and α1-Syntrophin Mediated by PDZ Domains	preclinical
Brinholi, F. F.-//-Farias, C. C.-//-Bonifácio, K. L.-//-Higachi, L.-//-Casagrande, R.-//-Moreira, E. G.-//-Barbosa, D. S.	Clozapine and olanzapine are better antioxidants than haloperidol, quetiapine, risperidone and ziprasidone in in vitro models	preclinical
Brunstein, M. G.-//-Ghisolfi, E. S.-//-Ramos, F. L.-//-Lara, D. R.	A clinical trial of adjuvant allopurinol therapy for moderately refractory schizophrenia	clinical
Buonaguro, E. F.-//-Tomasetti, C.-//-Chiodini, P.-//-Marmo, F.-//-Latte, G.-//-Rossi, R.-//-Avvisati, L.-//-Iasevoli, F.-//-de Bartolomeis, A.	Postsynaptic density protein transcripts are differentially modulated by minocycline alone or in add-on to haloperidol: Implications for treatment resistant schizophrenia	preclinical
Buosi, P.-//-Borghi, F. A.-//-Lopes, A. M.-//-Facincani, I. D. S.-//-Fernandes-Ferreira, R.-//-Oliveira-Brancati, C. I. F.-//-do Carmo, T. S.-//-Souza, D. R. S.-//-da Silva, D. G. H.-//-de Almeida, E. A.-//-de Araújo Filho, G. M.	Oxidative stress biomarkers in treatment-responsive and treatment-resistant schizophrenia patients	clinical
Burkhardt, C.-//-Kelly, J. P.-//-Lim, Y. H.-//-Filley, C. M.-//-Parker, W. D., Jr.	Neuroleptic medications inhibit complex I of the electron transport chain	preclinical
Cabungcal, J. H.-//-Steullet, P.-//-Morishita, H.-//-Kraftsik, R.-//-Cuenod, M.-//-Hensch, T. K.-//-Do, K. Q.	Perineuronal nets protect fast-spiking interneurons against oxidative stress	preclinical
Cabungcal, J. H.-//-Counotte, D. S.-//-Lewis, E.-//-Tejeda, H. A.-//-Piantadosi, P.-//-Pollock, C.-//-Calhoon, G. G.-//-Sullivan, E.-//-Presgraves, E.-//-Kil, J.-//-Hong, L. E.-//-Cuenod, M.-//-Do, K. Q.-//-O'Donnell, P.	Juvenile antioxidant treatment prevents adult deficits in a developmental model of schizophrenia	preclinical
Cabungcal, J. H.-//-Steullet, P.-//-Kraftsik, R.-//-Cuenod, M.-//-Do, K. Q.	A developmental redox dysregulation leads to spatio-temporal deficit of parvalbumin neuron circuitry in a schizophrenia mouse model	preclinical
Cardis, R.-//-Cabungcal, J. H.-//-Dwir, D.-//-Do, K. Q.-//-Steullet, P.	A lack of GluN2A-containing NMDA receptors confers a vulnerability to redox dysregulation: Consequences on parvalbumin interneurons, and their perineuronal nets	preclinical

Carmeli, C.-//-Knyazeva, M. G.-//-Cuénod, M.-//-Do, K. Q.	Glutathione precursor N-acetyl-cysteine modulates EEG synchronization in schizophrenia patients: a double-blind, randomized, placebo-controlled trial	clinical
Caruso, G.-//-Grasso, M.-//-Fidilio, A.-//-Tascedda, F.-//-Drago, F.-//-Caraci, F.	Antioxidant Properties of Second-Generation Antipsychotics: Focus on Microglia	review
Casademont, J.-//-Garrabou, G.-//-Miró, O.-//-López, S.-//-Pons, A.-//-Bernardo, M.-//-Cardellach, F.	Neuroleptic treatment effect on mitochondrial electron transport chain: peripheral blood mononuclear cells analysis in psychotic patients	clinical
Celano, E.-//-Tiraboschi, E.-//-Consogno, E.-//-D'Urso, G.-//-Mbakop, M. P.-//-Gennarelli, M.-//-de Bartolomeis, A.-//-Racagni, G.-//-Popoli, M.	Selective regulation of presynaptic calcium/calmodulin-dependent protein kinase II by psychotropic drugs	preclinical
Chan, S. T.-//-McCarthy, M. J.-//-Vawter, M. P.	Psychiatric drugs impact mitochondrial function in brain and other tissues	review
Chen, B. H.-//-Yan, B. C.-//-Park, J. H.-//-Ahn, J. H.-//-Lee, D. H.-//-Kim, I. H.-//-Cho, J. H.-//-Lee, J. C.-//-Kim, S. K.-//-Lee, B.-//-Cho, J. H.-//-Won, M. H.-//-Lee, Y. L.	Aripiprazole, an atypical antipsychotic drug, improves maturation and complexity of neuroblast dendrites in the mouse dentate gyrus via increasing superoxide dismutases	preclinical
Chen, H.-//-Detmer, S. A.-//-Ewald, A. J.-//-Griffin, E. E.-//-Fraser, S. E.-//-Chan, D. C.	Mitofusins Mfn1 and Mfn2 coordinately regulate mitochondrial fusion and are essential for embryonic development	preclinical
Chen, H.-//-McCaffery, J. M.-//-Chan, D. C.	Mitochondrial fusion protects against neurodegeneration in the cerebellum	preclinical
Choi, Y.-//-Chen, H. V.-//-Lipton, S. A.	Three pairs of cysteine residues mediate both redox and zn2+ modulation of the nmda receptor	preclinical
Chua, J. S.-//-Cowley, C. J.-//-Manavis, J.-//-Rofe, A. M.-//-Coyle, P.	Prenatal exposure to lipopolysaccharide results in neurodevelopmental damage that is ameliorated by zinc in mice	preclinical
Corcoba, A.-//-Steullet, P.-//-Duarte, J. M.-//-Van de Looij, Y.-//-Monin, A.-//-Cuenod, M.-//-Gruetter, R.-//-Do, K. Q.	Glutathione Deficit Affects the Integrity and Function of the Fimbria/Fornix and Anterior Commissure in Mice: Relevance for Schizophrenia	preclinical
Corti, C.-//-Xuereb, J. H.-//-Crepaldi, L.-//-Corsi, M.-//-Michielin, F.-//-Ferraguti, F.	Altered levels of glutamatergic receptors and Na+/K+ ATPase-α1 in the prefrontal cortex of subjects with schizophrenia	preclinical
Dakhale, G. N.-//-Khanzode, S. D.-//-Khanzode, S. S.-//-Saoji, A.	Supplementation of vitamin C with atypical antipsychotics reduces oxidative stress and improves the outcome of schizophrenia	clinical
Das, T. K.-//-Javadzadeh, A.-//-Dey, A.-//-Sabesan, P.-//-Théberge, J.-//-Radua, J.-//-Palaniyappan, L.	Antioxidant defense in schizophrenia and bipolar disorder: A meta-analysis of MRS studies of anterior cingulate glutathione	review
Davies, V. J.-//-Hollins, A. J.-//-Piechota, M. J.-//-Yip, W.-//-Davies, J. R.-//-White, K. E.-//-	Opa1 deficiency in a mouse model of autosomal dominant optic atrophy impairs mitochondrial morphology, optic nerve structure and visual function	preclinical

Nicols, P. P.-//-Boulton, M. E.-//-Votruba, M.		
de Bartolomeis, A.-//-Barone, A.-//-Vellucci, L.-//-Mazza, B.-//-Austin, M. C.-//-Iasevoli, F.-//-Ciccarelli, M.	Linking Inflammation, Aberrant Glutamate-Dopamine Interaction, and Post-synaptic Changes: Translational Relevance for Schizophrenia and Antipsychotic Treatment: a Systematic Review	review
De Lima, D. N., Jr.-//-Costa Filho, C. W. L.-//-Frota, I. J.-//-de Oliveira, A. L. B.-//-Menezes, C. E. S.-//-Chaves Filho, A. J. M.-//-Viana, G. A.-//-Campos, E. M.-//-Collares, M.-//-de Queiroz, M. G. R.-//-da Cruz Fonseca, S. G.-//-Vasconcelos, S. M. M.-//-Macêdo, D. S.-//-Sanders, L. L. O.	α-Lipoic Acid as Adjunctive Treatment for Schizophrenia: A Randomized Double-Blind Study	clinical
Devine, M. J.-//-Kittler, J. T.	Mitochondria at the neuronal presynapse in health and disease	review
Dickerson, F. B.-//-Stallings, C. R.-//-Origoni, A. E.-//-Sullens, A.-//-Khushalani, S.-//-Sandson, N.-//-Yolken, R. H.	A double-blind trial of adjunctive allopurinol for schizophrenia	clinical
Dietrich-Muszalska, A.-//-Kopka, J.-//-Kwiatkowska, A.	The effects of ziprasidone, clozapine and haloperidol on lipid peroxidation in human plasma (in vitro): comparison	clinical
Dietrich-Muszalska, A.-//-Kolińska-Łukaszuk, J.	Comparative effects of aripiprazole and selected antipsychotic drugs on lipid peroxidation in plasma	clinical
Do, K. Q.-//-Trabesinger, A. H.-//-Kirsten-Krüger, M.-//-Lauer, C. J.-//-Dydak, U.-//-Hell, D.-//-Holsboer, F.-//-Boesiger, P.-//-Cuénod, M.	Schizophrenia: glutathione deficit in cerebrospinal fluid and prefrontal cortex in vivo	clinical
Do, K. Q.-//-Cabungcal, J. H.-//-Frank, A.-//-Steullet, P.-//-Cuenod, M.	Redox dysregulation, neurodevelopment, and schizophrenia	review
Do, K. Q.-//-Cuenod, M.-//-Hensch, T. K.	Targeting Oxidative Stress and Aberrant Critical Period Plasticity in the Developmental Trajectory to Schizophrenia	review
Dorevitch, A.-//-Kalian, M.-//-Shlafman, M.-//-Lerner, V.	Treatment of long-term tardive dyskinesia with vitamin E	clinical
Doruk, A.-//-Uzun, O.-//-Ozşahin, A.	A placebo-controlled study of extract of ginkgo biloba added to clozapine in patients with treatment-resistant schizophrenia	clinical
Elimadi, A.-//-Bouillot, L.-//-Sapena, R.-//-Tillement, J. P.-//-Morin, D.	Dose-related inversion of cinnarizine and flunarizine effects on mitochondrial permeability transition	preclinical
Elkashef, A. M.-//-Al-Barazi, H.-//-Venable, D.-//-Baker, I.-//-Hill, J.-//-Apud, J.-//-Wyatt, R. J.	Dopamine effect on the mitochondria potential in B lymphocytes of schizophrenic patients and normal controls	preclinical
Elmorsy, E.-//-Alelwani, W.-//-Kattan, S.-//-Babteen, N.-//-Alnajeebi, A.-//-Ghulam, J.-//-Mosad, S.	Antipsychotics inhibit the mitochondrial bioenergetics of pancreatic beta cells isolated from CD1 mice	preclinical

Ertürk, A.-//-Wang, Y.-//-Sheng, M.	Local pruning of dendrites and spines by caspase-3-dependent and proteasome-limited mechanisms	preclinical
Farokhnia, M.-//-Azarkolah, A.-//-Adinehfar, F.-//-Khodaie-Ardakani, M. R.-//-Hosseini, S. M.-//-Yekehtaz, H.-//-Tabrizi, M.-//-Rezaei, F.-//-Salehi, B.-//-Sadeghi, S. M.-//-Moghadam, M.-//-Gharibi, F.-//-Mirshafiee, O.-//-Akhondzadeh, S.	N-acetylcysteine as an adjunct to risperidone for treatment of negative symptoms in patients with chronic schizophrenia: a randomized, double-blind, placebo-controlled study	clinical
Fernandez-Fernandez, S.-//-Bobo-Jimenez, V.-//-Requejo-Aguilar, R.-//-Gonzalez-Fernandez, S.-//-Resch, M.-//-Carabias-Carrasco, M.-//-Ros, J.-//-Almeida, A.-//-Bolaños, J. P.	Hippocampal neurons require a large pool of glutathione to sustain dendrite integrity and cognitive function	preclinical
Ferraris, D.-//-Duvall, B.-//-Ko, Y. S.-//-Thomas, A. G.-//-Rojas, C.-//-Majer, P.-//-Hashimoto, K.-//-Tsukamoto, T.	Synthesis and biological evaluation of D-amino acid oxidase inhibitors	preclinical
Flippo, K. H.-//-Strack, S.	An emerging role for mitochondrial dynamics in schizophrenia	review
Forder, J. P.-//-Tymianski, M.	Postsynaptic mechanisms of excitotoxicity: Involvement of postsynaptic density proteins, radicals, and oxidant molecules	review
Freudenberg, F.-//-Alttoa, A.-//-Reif, A.	Neuronal nitric oxide synthase (NOS1) and its adaptor, NOS1AP, as a genetic risk factors for psychiatric disorders	review
Frey, B. N.-//-Valvassori, S. S.-//-Gomes, K. M.-//-Martins, M. R.-//-Dal-Pizzol, F.-//-Kapczinski, F.-//-Quevedo, J.	Increased oxidative stress in submitochondrial particles after chronic amphetamine exposure	preclinical
Freyberg, Z.-//-Ferrando, S. J.-//-Javitch, J. A.	Roles of the Akt/GSK-3 and Wnt signaling pathways in schizophrenia and antipsychotic drug action	review
Gassó, P.-//-Mas, S.-//-Molina, O.-//-Bernardo, M.-//-Lafuente, A.-//-Parellada, E.	Neurotoxic/neuroprotective activity of haloperidol, risperidone and paliperidone in neuroblastoma cells	preclinical
Goh, X. X.-//-Tang, P. Y.-//-Tee, S. F.	Effects of antipsychotics on antioxidant defence system in patients with schizophrenia: A meta-analysis	review
Górny, M.-//-Bilska-Wilkosz, A.-//-Iciek, M.-//-Hereta, M.-//-Kamińska, K.-//-Kamińska, A.-//-Chwatko, G.-//-Rogóż, Z.-//-Lorenc-Koci, E.	Alterations in the Antioxidant Enzyme Activities in the Neurodevelopmental Rat Model of Schizophrenia Induced by Glutathione Deficiency during Early Postnatal Life	preclinical
Grima, G.-//-Benz, B.-//-Parpura, V.-//-Cuénod, M.-//-Do, K. Q.	Dopamine-induced oxidative stress in neurons with glutathione deficit: implication for schizophrenia	preclinical
Hashimoto, K.-//-Fujita, Y.-//-Horio, M.-//-Kunitachi, S.-//-Iyo,	Co-administration of a D-amino acid oxidase inhibitor potentiates the efficacy of D-serine in	preclinical

M.-//-Ferraris, D.-//-Tsukamoto, T.	attenuating prepulse inhibition deficits after administration of dizocilpine	
Hendouei, N.-//-Farnia, S.-//-Mohseni, F.-//-Salehi, A.-//-Bagheri, M.-//-Shadfar, F.-//-Barzegar, F.-//-Hoseini, S. D.-//-Charati, J. Y.-//-Shaki, F.	Alterations in oxidative stress markers and its correlation with clinical findings in schizophrenic patients consuming perphenazine, clozapine and risperidone	clinical
Hirata, Y.-//-Oka, K.-//-Yamamoto, S.-//-Watanabe, H.-//-Oh-Hashi, K.-//-Hirayama, T.-//-Nagasawa, H.-//-Takemori, H.-//-Furuta, K.	Haloperidol Prevents Oxytosis/Ferroptosis by Targeting Lysosomal Ferrous Ions in a Manner Independent of Dopamine D2 and Sigma-1 Receptors	preclinical
Huang, C. H.-//-Fu, S. H.-//-Hsu, S.-//-Huang, Y. Y.-//-Chen, S. T.-//-Hsu, B. R.	High-fat diet aggravates islet beta-cell toxicity in mice treated with clozapine	preclinical
Iasevoli, F.-//-Buonaguro, E. F.-//-Avagliano, C.-//-Barone, A.-//-Eramo, A.-//-Vellucci, L.-//-de Bartolomeis, A.	The Effects of Antipsychotics on the Synaptic Plasticity Gene Homer1a Depend on a Combination of Their Receptor Profile, Dose, Duration of Treatment, and Brain Regions Targeted	preclinical
Iwata, Y.-//-Nakajima, S.-//-Plitman, E.-//-Truong, P.-//-Bani-Fatemi, A.-//-Caravaggio, F.-//-Kim, J.-//-Shah, P.-//-Mar, W.-//-Chavez, S.-//-Remington, G.-//-Gerretsen, P.-//-De Luca, V.-//-Sailasuta, N.-//-Graff-Guerrero, A.	Glutathione Levels and Glutathione-Glutamate Correlation in Patients With Treatment-Resistant Schizophrenia	clinical
Jahan, M. S.-//-Tsuzuki, T.-//-Ito, T.-//-Bhuiyan, M. E. R.-//-Takahashi, I.-//-Takamatsu, H.-//-Kumanogoh, A.-//-Negishi, T.-//-Yukawa, K.	PlexinA1-deficient mice exhibit decreased cell density and augmented oxidative stress in parvalbumin-expressing interneurons in the medial prefrontal cortex	preclinical
Ji, B.-//-La, Y.-//-Gao, L.-//-Zhu, H.-//-Tian, N.-//-Zhang, M.-//-Yang, Y.-//-Zhao, X.-//-Tang, R.-//-Ma, G.-//-Zhou, J.-//-Meng, J.-//-Ma, J.-//-Zhang, Z.-//-Li, H.-//-Feng, G.-//-Wang, Y.-//-He, L.-//-Wan, C.	A comparative proteomics analysis of rat mitochondria from the cerebral cortex and hippocampus in response to antipsychotic medications	preclinical
Jiao, S.-//-Cao, T.-//-Cai, H.	Peripheral biomarkers of treatment-resistant schizophrenia: Genetic, inflammation and stress perspectives	review
Kilanczyk, E.-//-Saraswat Ohri, S.-//-Whittemore, S. R.-//-Hetman, M.	Antioxidant Protection of NADPH-Depleted Oligodendrocyte Precursor Cells Is Dependent on Supply of Reduced Glutathione	preclinical
Kim, H. K.-//-Andreazza, A. C.	The relationship between oxidative stress and post-translational modification of the dopamine transporter in bipolar disorder	review
Kim, Y.-//-Vadodaria, K. C.-//-Lenkei, Z.-//-Kato, T.-//-Gage, F.	Mitochondria, Metabolism, and Redox Mechanisms in Psychiatric Disorders	review

H.-//-Marchetto, M. C.-//-Santos, R.		
Klauser, P.-//-Xin, L.-//-Fournier, M.-//-Griffa, A.-//-Cleusix, M.-//-Jenni, R.-//-Cuenod, M.-//-Gruetter, R.-//-Hagmann, P.-//-Conus, P.-//-Baumann, P. S.-//-Do, K. Q.	N-acetylcysteine add-on treatment leads to an improvement of fornix white matter integrity in early psychosis: a double-blind randomized placebo-controlled trial	clinical
Kung, L.-//-Roberts, R. C.	Mitochondrial pathology in human schizophrenic striatum: a postmortem ultrastructural study	preclinical
Kvajo, M.-//-Dhilla, A.-//-Swor, D. E.-//-Karayiorgou, M.-//-Gogos, J. A.	Evidence implicating the candidate schizophrenia/bipolar disorder susceptibility gene G72 in mitochondrial function	preclinical
Kwon, D. J.-//-Ju, S. M.-//-Youn, G. S.-//-Choi, S. Y.-//-Park, J.	Suppression of iNOS and COX-2 expression by flavokawain A via blockade of NF-κB and AP-1 activation in RAW 264.7 macrophages	preclinical
Lavoie, S.-//-Murray, M. M.-//-Deppen, P.-//-Knyazeva, M. G.-//-Berk, M.-//-Boulat, O.-//-Bovet, P.-//-Bush, A. I.-//-Conus, P.-//-Copolov, D.-//-Fornari, E.-//-Meuli, R.-//-Solida, A.-//-Vianin, P.-//-Cuénod, M.-//-Buclin, T.-//-Do, K. Q.	Glutathione precursor, N-acetyl-cysteine, improves mismatch negativity in schizophrenia patients	clinical
Lech, M. A.-//-Leśkiewicz, M.-//-Kamińska, K.-//-Rogóż, Z.-//-Lorenc-Koci, E.	Glutathione Deficiency during Early Postnatal Development Causes Schizophrenia-Like Symptoms and a Reduction in BDNF Levels in the Cortex and Hippocampus of Adult Sprague-Dawley Rats	preclinical
Limongi, R.-//-Jeon, P.-//-Théberge, J.-//-Palaniyappan, L.	Counteracting Effects of Glutathione on the Glutamate-Driven Excitation/Inhibition Imbalance in First-Episode Schizophrenia: A 7T MRS and Dynamic Causal Modeling Study	clinical
Liu, H.-//-Liu, H.-//-Jiang, S.-//-Su, L.-//-Lu, Y.-//-Chen, Z.-//-Li, X.-//-Li, X.-//-Wang, X.-//-Xiu, M.-//-Zhang, X.	Sex-Specific Association between Antioxidant Defense System and Therapeutic Response to Risperidone in Schizophrenia: A Prospective Longitudinal Study	clinical
Liu, H.-//-Yu, R.-//-Gao, Y.-//-Li, X.-//-Guan, X.-//-Thomas, K.-//-Xiu, M.-//-Zhang, X.	Antioxidant Enzymes and Weight Gain in Drug-naive First-episode Schizophrenia Patients Treated with Risperidone for 12 Weeks: A Prospective Longitudinal Study	clinical
Lopes-Rocha, A.-//-Bezerra, T. O.-//-Zanotto, R.-//-Lages Nascimento, I.-//-Rodrigues, A.-//-Salum, C.	The Antioxidant N-Acetyl-L-Cysteine Restores the Behavioral Deficits in a Neurodevelopmental Model of Schizophrenia Through a Mechanism That Involves Nitric Oxide	preclinical
Maas, D. A.-//-Eijsink, V. D.-//-van Hulten, J. A.-//-Panic, R.-//-De Weerd, P.-//-Homberg, J. R.-//-Vallès, A.-//-Nait-Oumesmar, B.-//-Martens, G. J. M.	Antioxidant treatment ameliorates prefrontal hypomyelination and cognitive deficits in a rat model of schizophrenia	preclinical

MacDowell, K. S.-//-Martín-Hernández, D.-//-Ulecia-Morón, C.-//-Bris Á, G.-//-Madrigal, J. L. M.-//-García-Bueno, B.-//-Caso, J. R.	Paliperidone attenuates chronic stress-induced changes in the expression of inflammasomes-related protein in the frontal cortex of male rats	preclinical
Magalhães, P. V.-//-Dean, O.-//-Andreazza, A. C.-//-Berk, M.-//-Kapczinski, F.	Antioxidant treatments for schizophrenia	review
Marchbanks, R. M.-//-Ryan, M.-//-Day, I. N.-//-Owen, M.-//-McGuffin, P.-//-Whatley, S. A.	A mitochondrial DNA sequence variant associated with schizophrenia and oxidative stress	preclinical
Marí, M.-//-Morales, A.-//-Colell, A.-//-García-Ruiz, C.-//-Fernández-Checa, J. C.	Mitochondrial glutathione, a key survival antioxidant	review
Masoud, S. T.-//-Vecchio, L. M.-//-Bergeron, Y.-//-Hossain, M. M.-//-Nguyen, L. T.-//-Bermejo, M. K.-//-Kile, B.-//-Sotnikova, T. D.-//-Siesser, W. B.-//-Gainetdinov, R. R.-//-Wightman, R. M.-//-Caron, M. G.-//-Richardson, J. R.-//-Miller, G. W.-//-Ramsey, A. J.-//-Cyr, M.-//-Salahpour, A.	Increased expression of the dopamine transporter leads to loss of dopamine neurons, oxidative stress and l-DOPA reversible motor deficits	preclinical
Maurer, I.-//-Möller, H. J.	Inhibition of complex I by neuroleptics in normal human brain cortex parallels the extrapyramidal toxicity of neuroleptics	preclinical
Maurer, I.-//-Zierz, S.-//-Möller, H.	Evidence for a mitochondrial oxidative phosphorylation defect in brains from patients with schizophrenia	preclinical
Meade, A. J.-//-Meloni, B. P.-//-Cross, J.-//-Bakker, A. J.-//-Fear, M. W.-//-Mastaglia, F. L.-//-Watt, P. M.-//-Knuckey, N. W.	AP-1 inhibitory peptides are neuroprotective following acute glutamate excitotoxicity in primary cortical neuronal cultures	preclinical
Millar, J. K.-//-James, R.-//-Christie, S.-//-Porteous, D. J.	Disrupted in schizophrenia 1 (DISC1): subcellular targeting and induction of ring mitochondria	preclinical
Mishra, A.-//-Reeta, K. H.-//-Sarangi, S. C.-//-Maiti, R.-//-Sood, M.	Effect of add-on alpha lipoic acid on psychopathology in patients with treatment-resistant schizophrenia: a pilot randomized double-blind placebo-controlled trial	clinical
Monin, A.-//-Baumann, P. S.-//-Griffa, A.-//-Xin, L.-//-Mekle, R.-//-Fournier, M.-//-Butticaz, C.-//-Klaey, M.-//-Cabungcal, J. H.-//-Steullet, P.-//-Ferrari, C.-//-Cuenod, M.-//-Gruetter, R.-//-Thiran, J. P.-//-Hagmann, P.-//-Conus, P.-//-Do, K. Q.	Glutathione deficit impairs myelin maturation: relevance for white matter integrity in schizophrenia patients	clinical
Mullier, E.-//-Roine, T.-//-Griffa, A.-//-Xin, L.-//-Baumann, P. S.-//-Klauser, P.-//-Cleusix, M.-//-Jenni, R.-//-Alemàn-Gómez, Y.-	N-Acetyl-Cysteine Supplementation Improves Functional Connectivity Within the Cingulate Cortex in Early Psychosis: A Pilot Study	clinical

//-Gruetter, R.-//-Conus, P.-//-Do, K. Q.-//-Hagmann, P.		
Munakata, K.-//-Iwamoto, K.-//-Bundo, M.-//-Kato, T.	Mitochondrial DNA 3243A>G mutation and increased expression of LARS2 gene in the brains of patients with bipolar disorder and schizophrenia	preclinical
Musazzi, L.-//-Treccani, G.-//-Popoli, M.	Functional and structural remodeling of glutamate synapses in prefrontal and frontal cortex induced by behavioral stress	review
Myken, A. N.-//-Ebdrup, B. H.-//-Sørensen, M. E.-//-Broberg, B. V.-//-Skjerbæk, M. W.-//-Glenthøj, B. Y.-//-Lykkesfeldt, J.-//-Nielsen, MØ	Lower Vitamin C Levels Are Associated With Less Improvement in Negative Symptoms in Initially Antipsychotic-Naïve Patients With First-Episode Psychosis	clinical
Naoi, Makoto-//-Maruyama, Wakako-//-Shamoto-Nagai, Masayo	Neuroprotective Function of Rasagiline and Selegiline, Inhibitors of Type B Monoamine Oxidase, and Role of Monoamine Oxidases in Synucleinopathies	review
Neill, E.-//-Rossell, S. L.-//-Yolland, C.-//-Meyer, D.-//-Galletly, C.-//-Harris, A.-//-Siskind, D.-//-Berk, M.-//-Bozaoglu, K.-//-Dark, F.-//-Dean, O. M.-//-Francis, P. S.-//-Liu, D.-//-Phillipou, A.-//-Sarris, J.-//-Castle, D. J.	N-Acetylcysteine (NAC) in Schizophrenia Resistant to Clozapine: A Double-Blind, Randomized, Placebo-Controlled Trial Targeting Negative Symptoms	clinical
Nepliouev, I.-//-Zhang, Z. S.-//-Stiber, J. A.	Effect of oxidative stress on homer scaffolding proteins	preclinical
Ninan, I.-//-Jardemark, K. E.-//-Liang, X.-//-Wang, R. Y.	Calcium/calmodulin-dependent kinase II is involved in the facilitating effect of clozapine on NMDA- and electrically evoked responses in the medial prefrontal cortical pyramidal cells	preclinical
Norkett, R.-//-Modi, S.-//-Birsa, N.-//-Atkin, T. A.-//-Ivankovic, D.-//-Pathania, M.-//-Trossbach, S. V.-//-Korth, C.-//-Hirst, W. D.-//-Kittler, J. T.	DISC1-dependent Regulation of Mitochondrial Dynamics Controls the Morphogenesis of Complex Neuronal Dendrites	preclinical
Page, M. J.-//-McKenzie, J. E.-//-Bossuyt, P. M.-//-Boutron, I.-//-Hoffmann, T. C.-//-Mulrow, C. D.-//-Shamseer, L.-//-Tetzlaff, J. M.-//-Akl, E. A.-//-Brennan, S. E.-//-Chou, R.-//-Glanville, J.-//-Grimshaw, J. M.-//-Hróbjartsson, A.-//-Lalu, M. M.-//-Li, T.-//-Loder, E. W.-//-Mayo-Wilson, E.-//-McDonald, S.-//-McGuinness, L. A.-//-Stewart, L. A.-//-Thomas, J.-//-Tricco, A. C.-//-Welch, V. A.-//-Whiting, P.-//-Moher, D.	The PRISMA 2020 statement: an updated guideline for reporting systematic reviews	review
Palaniyappan, L.-//-Park, M. T. M.-//-Jeon, P.-//-Limongi, R.-//-	Is There a Glutathione Centered Redox Dysregulation Subtype of Schizophrenia?	review

Yang, K.-//-Sawa, A.-//-Théberge, J.		
Pandurangi, A. K.-//-Buckley, P. F.	Inflammation, Antipsychotic Drugs, and Evidence for Effectiveness of Anti-inflammatory Agents in Schizophrenia	review
Park, S. U.-//-Ferrer, J. V.-//-Javitch, J. A.-//-Kuhn, D. M.	Peroxynitrite inactivates the human dopamine transporter by modification of cysteine 342: potential mechanism of neurotoxicity in dopamine neurons	preclinical
Pedrini, M.-//-Massuda, R.-//-Fries, G. R.-//-de Bittencourt Pasquali, M. A.-//-Schnorr, C. E.-//-Moreira, J. C.-//-Teixeira, A. L.-//-Lobato, M. I.-//-Walz, J. C.-//-Belmonte-de-Abreu, P. S.-//-Kauer-Sant'Anna, M.-//-Kapczinski, F.-//-Gama, C. S.	Similarities in serum oxidative stress markers and inflammatory cytokines in patients with overt schizophrenia at early and late stages of chronicity	clinical
Perez-Cruz, C.-//-Müller-Keuker, J. I.-//-Heilbronner, U.-//-Fuchs, E.-//-Flügge, G.	Morphology of pyramidal neurons in the rat prefrontal cortex: lateralized dendritic remodeling by chronic stress	preclinical
Phensy, A.-//-Duzdabanian, H. E.-//-Brewer, S.-//-Panjabi, A.-//-Driskill, C.-//-Berz, A.-//-Peng, G.-//-Kroener, S.	Antioxidant Treatment with N-acetyl Cysteine Prevents the Development of Cognitive and Social Behavioral Deficits that Result from Perinatal Ketamine Treatment	preclinical
Pillai, A.-//-Veeranan-Karmegam, R.-//-Dhandapani, K. M.-//-Mahadik, S. P.	Cystamine prevents haloperidol-induced decrease of BDNF/TrkB signaling in mouse frontal cortex	preclinical
Pillinger, T.-//-D'Ambrosio, E.-//-McCutcheon, R.-//-Howes, O. D.	Is psychosis a multisystem disorder? A meta-review of central nervous system, immune, cardiometabolic, and endocrine alterations in first-episode psychosis and perspective on potential models	review
Prince, J. A.-//-Yassin, M. S.-//-Oreland, L.	Neuroleptic-induced mitochondrial enzyme alterations in the rat brain	preclinical
Rambaud, V.-//-Marzo, A.-//-Chaumette, B.	Oxidative Stress and Emergence of Psychosis	review
Rapado-Castro, M.-//-Berk, M.-//-Venugopal, K.-//-Bush, A. I.-//-Dodd, S.-//-Dean, O. M.	Towards stage specific treatments: effects of duration of illness on therapeutic response to adjunctive treatment with N-acetyl cysteine in schizophrenia	clinical
Rapado-Castro, M.-//-Dodd, S.-//-Bush, A. I.-//-Malhi, G. S.-//-Skvarc, D. R.-//-On, Z. X.-//-Berk, M.-//-Dean, O. M.	Cognitive effects of adjunctive N-acetyl cysteine in psychosis	clinical
Raudenska, M.-//-Gumulec, J.-//-Babula, P.-//-Stracina, T.-//-Sztalmachova, M.-//-Polanska, H.-//-Adam, V.-//-Kizek, R.-//-Novakova, M.-//-Masarik, M.	Haloperidol cytotoxicity and its relation to oxidative stress	review
Rice, M. W.-//-Smith, K. L.-//-Roberts, R. C.-//-Perez-Costas, E.-//-Melendez-Ferro, M.	Assessment of cytochrome C oxidase dysfunction in the substantia nigra/ventral tegmental area in schizophrenia	preclinical

Rollins, B. L.-//-Morgan, L.-//-Hjelm, B. E.-//-Sequeira, A.-//-Schatzberg, A. F.-//-Barchas, J. D.-//-Lee, F. S.-//-Myers, R. M.-//-Watson, S. J.-//-Akil, H.-//-Potkin, S. G.-//-Bunney, W. E.-//-Vawter, M. P.	Mitochondrial Complex I Deficiency in Schizophrenia and Bipolar Disorder and Medication Influence	preclinical
Rosenfeld, M.-//-Brenner-Lavie, H.-//-Ari, S. G.-//-Kavushansky, A.-//-Ben-Shachar, D.	Perturbation in mitochondrial network dynamics and in complex I dependent cellular respiration in schizophrenia	preclinical
Rushlow, W. J.-//-Seah, C.-//-Sutton, L. P.-//-Bjelica, A.-//-Rajakumar, N.	Antipsychotics affect multiple calcium calmodulin dependent proteins	preclinical
Sacchi, S.-//-Novellis, V.-//-Paolone, G.-//-Nuzzo, T.-//-Iannotta, M.-//-Belardo, C.-//-Squillace, M.-//-Bolognesi, P.-//-Rosini, E.-//-Motta, Z.-//-Frassineti, M.-//-Bertolino, A.-//-Pollegioni, L.-//-Morari, M.-//-Maione, S.-//-Errico, F.-//-Usiello, A.	Olanzapine, but not clozapine, increases glutamate release in the prefrontal cortex of freely moving mice by inhibiting D-aspartate oxidase activity	preclinical
Sagara, Y.	Induction of reactive oxygen species in neurons by haloperidol	preclinical
Scaini, G.-//-Quevedo, J.-//-Velligan, D.-//-Roberts, D. L.-//-Raventos, H.-//-Walss-Bass, C.	Second generation antipsychotic-induced mitochondrial alterations: Implications for increased risk of metabolic syndrome in patients with schizophrenia	preclinical
Schumacher, J.-//-Jamra, R. A.-//-Freudenberg, J.-//-Becker, T.-//-Ohlraun, S.-//-Otte, A. C.-//-Tullius, M.-//-Kovalenko, S.-//-Bogaert, A. V.-//-Maier, W.-//-Rietschel, M.-//-Propping, P.-//-Nöthen, M. M.-//-Cichon, S.	Examination of G72 and D-amino-acid oxidase as genetic risk factors for schizophrenia and bipolar affective disorder	preclinical
Semenovich, D. S.-//-Plotnikov, E. Y.-//-Titko, O. V.-//-Lukiyenko, E. P.-//-Kanunnikova, N. P.	Effects of Panthenol and N-Acetylcysteine on Changes in the Redox State of Brain Mitochondria under Oxidative Stress In Vitro	preclinical
Sepehrmanesh, Z.-//-Heidary, M.-//-Akasheh, N.-//-Akbari, H.-//-Heidary, M.	Therapeutic effect of adjunctive N-acetyl cysteine (NAC) on symptoms of chronic schizophrenia: A double-blind, randomized clinical trial	clinical
Seybolt, S. E.	Is it time to reassess alpha lipoic acid and niacinamide therapy in schizophrenia?	review
Singh, O. P.-//-Chakraborty, I.-//-Dasgupta, A.-//-Datta, S.	A comparative study of oxidative stress and interrelationship of important antioxidants in haloperidol and olanzapine treated patients suffering from schizophrenia	clinical
Smith, G. A.-//-Lin, T. H.-//-Sheehan, A. E.-//-Van der Goes van Naters, W.-//-Neukomm, L. J.-//-Graves, H. K.-//-Bis-Brewer,	Glutathione S-Transferase Regulates Mitochondrial Populations in Axons through Increased Glutathione Oxidation	preclinical

D. M.-//-Züchner, S.-//-Freeman, M. R.		
Somerville, S. M.-//-Lahti, A. C.-//-Conley, R. R.-//-Roberts, R. C.	Mitochondria in the striatum of subjects with schizophrenia: relationship to treatment response	preclinical
Subramanyam, B.-//-Rollema, H.-//-Woolf, T.-//-Castagnoli, N., Jr.	Identification of a potentially neurotoxic pyridinium metabolite of haloperidol in rats	preclinical
Sun, J.-//-Zhang, X.-//-Cong, Q.-//-Chen, D.-//-Yi, Z.-//-Huang, H.-//-Wang, C.-//-Li, M.-//-Zeng, R.-//-Liu, Y.-//-Huai, C.-//-Chen, L.-//-Liu, C.-//-Zhang, Y.-//-Xu, Y.-//-Fan, L.-//-Wang, G.-//-Song, C.-//-Wei, M.-//-Du, H.-//-Zhu, J.-//-He, L.-//-Qin, S.	miR143-3p-Mediated NRG-1-Dependent Mitochondrial Dysfunction Contributes to Olanzapine Resistance in Refractory Schizophrenia	preclinical
Svane, K. C.-//-Asis, E. K.-//-Omelchenko, A.-//-Kunnath, A. J.-//-Brzustowicz, L. M.-//-Silverstein, S. M.-//-Firestein, B. L.	d-Serine administration affects nitric oxide synthase 1 adaptor protein and DISC1 expression in sex-specific manner	preclinical
Swanepoel, T.-//-Möller, M.-//-Harvey, B. H.	N-acetyl cysteine reverses bio-behavioural changes induced by prenatal inflammation, adolescent methamphetamine exposure and combined challenges	preclinical
Tsugawa, S.-//-Noda, Y.-//-Tarumi, R.-//-Mimura, Y.-//-Yoshida, K.-//-Iwata, Y.-//-Elsalhy, M.-//-Kuromiya, M.-//-Kurose, S.-//-Masuda, F.-//-Morita, S.-//-Ogyu, K.-//-Plitman, E.-//-Wada, M.-//-Miyazaki, T.-//-Graff-Guerrero, A.-//-Mimura, M.-//-Nakajima, S.	Glutathione levels and activities of glutathione metabolism enzymes in patients with schizophrenia: A systematic review and meta-analysis	review
Ueno, H.-//-Nishigaki, Y.-//-Kong, Q. P.-//-Fuku, N.-//-Kojima, S.-//-Iwata, N.-//-Ozaki, N.-//-Tanaka, M.	Analysis of mitochondrial DNA variants in Japanese patients with schizophrenia	preclinical
Uludag, K.-//-Wang, D. M.-//-Zhang, X. Y.	Tardive Dyskinesia Development, Superoxide Dismutase Levels, and Relevant Genetic Polymorphisms	review
Unsal, C.-//-Albayrak, Y.-//-Albayrak, N.-//-Kuloglu, M.-//-Hashimoto, K.	Reduced serum paraoxonase 1 (PON1) activity in patients with schizophrenia treated with olanzapine but not quetiapine	clinical
Wąsik, A.-//-Białoń, M.-//-Żarnowska, M.-//-Antkiewicz-Michaluk, L.	Comparison of the effects of 1MeTIQ and olanzapine on performance in the elevated plus maze test and monoamine metabolism in the brain after ketamine treatment	preclinical
Wei, Z.-//-Bai, O.-//-Richardson, J. S.-//-Mousseau, D. D.-//-Li, X. M.	Olanzapine protects PC12 cells from oxidative stress induced by hydrogen peroxide	preclinical

Weiden, P. J.-//-Breier, A.-//-Kavanagh, S.-//-Miller, A. C.-//-Brannan, S. K.-//-Paul, S. M.	Antipsychotic Efficacy of KarXT (Xanomeline-Trospium): Post Hoc Analysis of Positive and Negative Syndrome Scale Categorical Response Rates, Time Course of Response, and Symptom Domains of Response in a Phase 2 Study	clinical
Weiser, M.-//-Gershon, A. A.-//-Rubinstein, K.-//-Petcu, C.-//-Ladea, M.-//-Sima, D.-//-Podea, D.-//-Keefe, R. S.-//-Davis, J. M.	A randomized controlled trial of allopurinol vs. placebo added on to antipsychotics in patients with schizophrenia or schizoaffective disorder	clinical
Weng, Yu-Ting-//-Chien, Ting-//-Kuan, I. I.-//-Chern, Yijuang	The TRAX, DISC1, and GSK3 complex in mental disorders and therapeutic interventions	review
Whatley, S. A.-//-Curti, D.-//-Das Gupta, F.-//-Ferrier, I. N.-//-Jones, S.-//-Taylor, C.-//-Marchbanks, R. M.	Superoxide, neuroleptics and the ubiquinone and cytochrome b5 reductases in brain and lymphocytes from normals and schizophrenic patients	preclinical
Whitehurst, T.-//-Howes, O.	The role of mitochondria in the pathophysiology of schizophrenia: A critical review of the evidence focusing on mitochondrial complex one	review
Wu, J.-//-Li, A.-//-Li, Y.-//-Li, X.-//-Zhang, Q.-//-Song, W.-//-Wang, Y.-//-Ogutu, J. O.-//-Wang, J.-//-Li, J.-//-Tang, R.-//-Zhang, F.	Chlorpromazine inhibits mitochondrial apoptotic pathway via increasing expression of tissue factor	preclinical
Xin, R.-//-Chen, Z.-//-Fu, J.-//-Shen, F.-//-Zhu, Q.-//-Huang, F.	Xanomeline Protects Cortical Cells From Oxygen-Glucose Deprivation via Inhibiting Oxidative Stress and Apoptosis	preclinical
Xuan, Y.-//-Yan, G.-//-Wu, R.-//-Huang, Q.-//-Li, X.-//-Xu, H.	The cuprizone-induced changes in (1)H-MRS metabolites and oxidative parameters in C57BL/6 mouse brain: Effects of quetiapine	preclinical
Yabuki, Y.-//-Wu, L.-//-Fukunaga, K.	Cognitive enhancer ST101 improves schizophrenia-like behaviors in neonatal ventral hippocampus-lesioned rats in association with improved CaMKII/PKC pathway	preclinical
Yang, M.-//-Wang, C.-//-Zhao, G.-//-Kong, D.-//-Liu, L.-//-Yuan, S.-//-Chen, W.-//-Feng, C.-//-Li, Z.	Comparative Analysis of the Pre- and Post-Medication Effects of Antipsychotic Agents on the Blood-Based Oxidative Stress Biomarkers in Patients with Schizophrenia: A Meta-Analysis	review
Zamani, E.-//-Ahmadi Shad, A.-//-Fatemi, H.-//-Mahboubi, S.-//-Motavallian, A.-//-Evazalipour, M.	Assessment of Protective Effects of Carvacrol on Haloperidol-Induced Oxidative Stress and Genotoxicity in Human Peripheral Blood Lymphocytes	clinical
Zerin Khan, Fatema-//-Sultana, Syeda Papia-//-Akhter, Nargis-//-Mosaddek, Abu Syed Md	Effect of Olanzapine and Risperidone on Oxidative Stress in Schizophrenia Patients %J International Biological and Biomedical Journal	clinical
Zhang, W. F.-//-Tan, Y. L.-//-Zhang, X. Y.-//-Chan, R. C.-//-Wu, H. R.-//-Zhou, D. F.	Extract of Ginkgo biloba treatment for tardive dyskinesia in schizophrenia: a randomized, double-blind, placebo-controlled trial	clinical
Zhang, X. Y.-//-Zhou, D. F.-//-Zhang, P. Y.-//-Wu, G. Y.-//-Su, J. M.-//-Cao, L. Y.	A double-blind, placebo-controlled trial of extract of Ginkgo biloba added to haloperidol in treatment-resistant patients with schizophrenia	clinical

Zhang, X. Y.-//-Zhou, D. F.-//-Cao, L. Y.-//-Zhang, P. Y.-//-Wu, G. Y.-//-Shen, Y. C.	The effect of risperidone treatment on superoxide dismutase in schizophrenia	clinical
Zhang, X. Y.-//-Zhou, D. F.-//-Cao, L. Y.-//-Xu, C. Q.-//-Chen, D. C.-//-Wu, G. Y.	The effect of vitamin E treatment on tardive dyskinesia and blood superoxide dismutase: a double-blind placebo-controlled trial	clinical
